# Supplementary material for: Hotspot propensity across mutational processes
Source: Mol Syst Biol. 2023 Dec 20;20(1):6–27. doi: 10.1038/s44320-023-00001-w (PMC10883281; doi:10.1038/s44320-023-00001-w)
Supplement: Supplementary file 1 — Appendix [file 44320_2023_1_MOESM1_ESM.pdf]

Appendix

Hotspot propensity across mutational processes

Authors

Claudia Arnedo-Pac[1,2], Ferran Muiños[1,2], Abel Gonzalez-Perez[1,2,#] & Nuria Lopez-Bigas[1,2,3,#]

Affiliations

[1] Institute for Research in Biomedicine (IRB Barcelona), The Barcelona Institute of Science and Technology, Barcelona, Spain.

[2] Centro de Investigación Biomédica en Red en Cáncer, Instituto de Salud Carlos III, Madrid, Spain.

[3] Institució Catalana de Recerca i Estudis Avançats (ICREA), Barcelona, Spain.

# To whom correspondence should be addressed.

Table of contents

Appendix Figures……………………………………………………………...3

Appendix Figure S1. Hotspot size across cancer types……………………………………………………3
Appendix Figure S2. Top recurrent hotspots and their distribution across the genome…………………..4
Appendix Figure S3. Frequency of hotspots formation across mutation types…………………………..6
Appendix Figure S4. Relationship between the increase of hotspot burden, mutation burden and sample size……………………………………………………………………………………………………....7
Appendix Figure S5. Relationship between observed hotspots and mutations……………………………8
Appendix Figure S6. Relationship between observed indel hotspots and mutations……………………..10
Appendix Figure S7. Conversion rates……………………………………………………………………12
Appendix Figure S8. Hotspots shared between cancer types………………………………………………13
Appendix Figure S9. Hotspot enrichment across six pyrimidine-based substitution types………………14
Appendix Figure S10. Hotspot enrichment across 96-based trinucleotide single base substitutions…...15
Appendix Figure S11. Hotspot enrichment across indel categories……………………………………...17
Appendix Figure S12. COSMIC reference signatures set decomposition of extracted SBS signatures……………………………..……………………………………………………………19
Appendix Figure S13. Proportion of hotspots per signature across cancers………………………………20
Appendix Figure S14. Hotspot logos across signatures…………………………………………………...21
Appendix Figure S15. Relationship between hotspot burden, mutation burden and active samples per signature……………………………………………………………………………………………………..22
Appendix Figure S16. Hotspot propensity with increased mutation burden……………………………...23
Appendix Figure S17. Signature frequency inside and outside hotspots………………………………...24
Appendix Figure S18. Hotspots overlapping genomic elements……………………………………………26
Appendix Figure S19. Relationship between hotspot burden and mutation burden across genome megabases…………………………………………………………………………………………………….28
Appendix Figure S20. Mutation rates across genomic regions with different chromatin accessibility…..30
Appendix Figure S21. Mutation rate across genomic regions with different replication timing…………..32
Appendix Figure S22. Mutation rate across genomic regions with different transcriptional activity……..34
Appendix Figure S23. Distribution of mutations across genome megabases……………………………..36
Appendix Figure S24. Expected hotspot propensity using submegabase models………………………..38
Appendix Figure S25. Expected hotspot propensity with larger subsampling……………………………..39
Appendix Figure S26. Hotspot propensity in Barrett's esophagus………………………………………….40

Appendix Notes………………………………………………………………..41

Appendix Note 1. HotspotFinder…………………………………………………………………………...41
Appendix Note 2. Mutational signatures extraction…………………………………………………………49
Appendix Note 3. Assignment of mutational signatures to mutations and hotspots………………………56
Appendix Note 4. Comment on hotspot propensity………………………………………………………...62
Appendix Note 5. Theoretical models of hotspot propensity………………………………………………65

Appendix References…………………………………………………………71

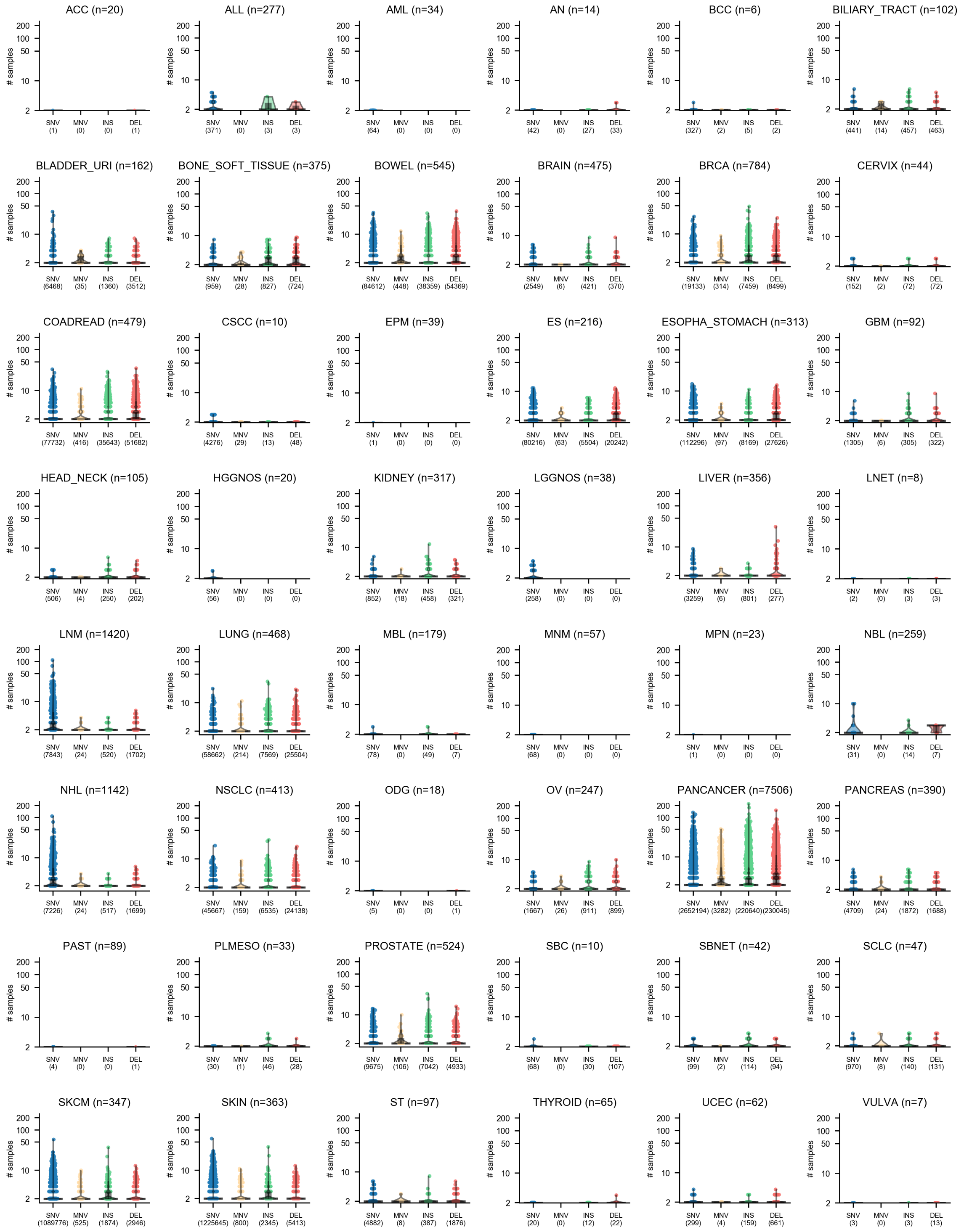

Appendix Figure S1. Hotspot size across cancer types. Distribution of hotspot sizes across the four mutation types (SNVs, MNVs, insertions and deletions) per cancer type. Dots represent individual hotspots per mutation type (total number is shown in parenthesis). Horizontal black lines show median hotspot size. Error bars show the range of the distribution.

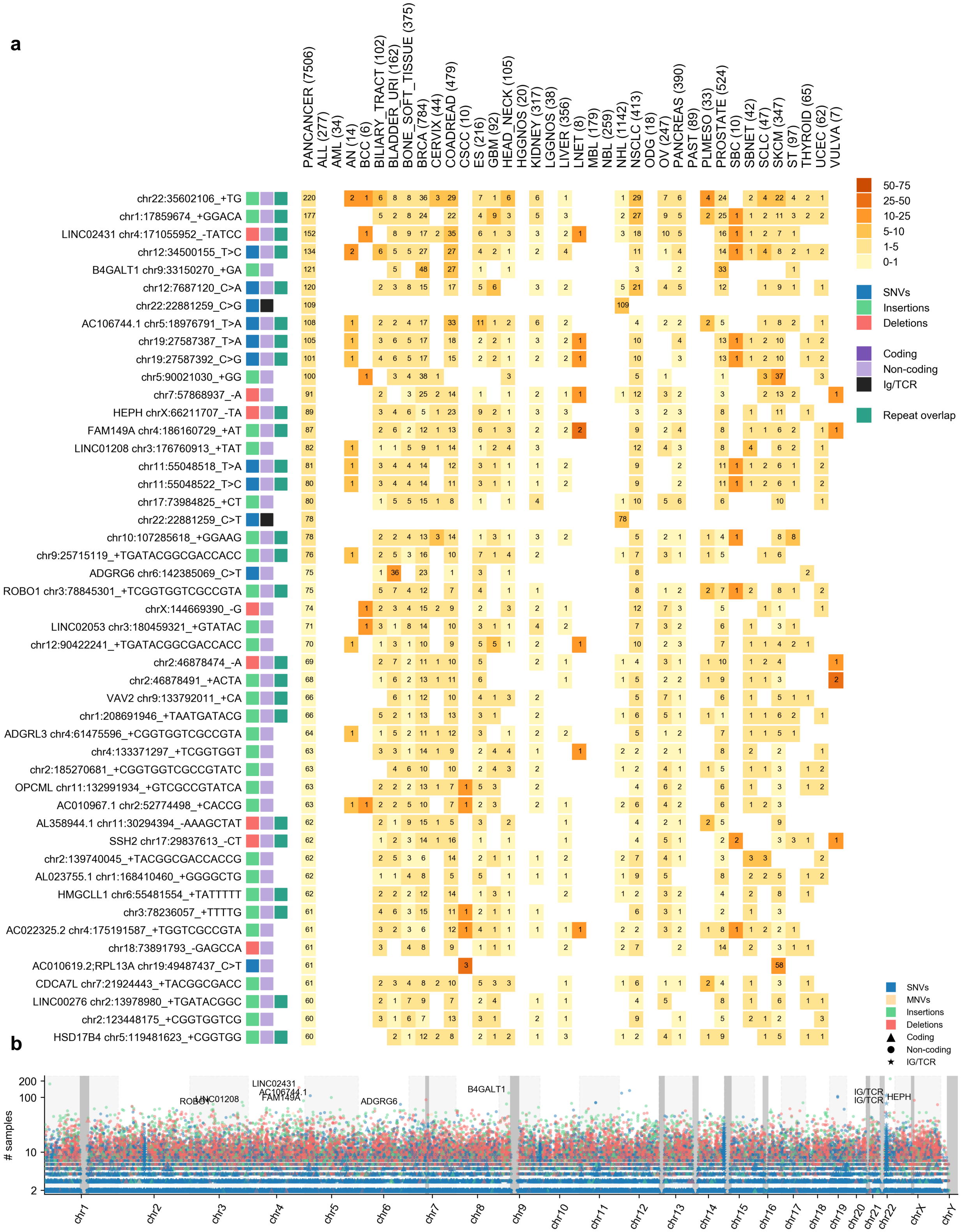
a
PANCANCER (7506)
ALL (277)
AML (34)
AN (14)
BCC (6)
BILIARY_TRACT (102)
BLADDER_URI (162)
BONE_SOFT_TISSUE (375)
BRCA (784)
CERVIX (44)
COADREAD (479)
CSCC (10)
ES (216)
GBM (92)
HEAD_NECK (105)
HGGNOS (20)
KIDNEY (317)
LGGNOS (38)
LIVER (356)
LNET (8)
MBL (179)
NBL (259)
NHL (1142)
NSCLC (413)
ODG (18)
OV (247)
PANCREAS (390)
PAST (89)
PLMESO (33)
PROSTATE (524)
SBC (10)
SBNET (42)
SCLC (47)
SKCM (347)
ST (97)
THYROID (65)
UCEC (62)
VULVA (7)
chr22:35602106_+TG
chr1:17859674_+GGACA
LINC02431 chr4:171055952_-TATCC
chr12:34500155_T>C
B4GALT1 chr9:33150270_+GA
chr12:7687120_C>A
chr22:22881259_C>G
AC106744.1 chr5:18976791_T>A
chr19:27587387_T>A
chr19:27587392_C>G
chr5:90021030_+GG
chr7:57868937_-A
HEPH chrX:66211707_-TA
FAM149A chr4:186160729_+AT
LINC01208 chr3:176760913_+TAT
chr11:55048518_T>A
chr11:55048522_T>C
chr17:73984825_+CT
chr22:22881259_C>T
chr10:107285618_+GGAAG
chr9:25715119_+TGATACGGCGACCACC
ADGRG6 chr6:142385069_C>T
ROBO1 chr3:78845301_+TCGGTGGTCGCCGTA
chrX:144669390_-G
LINC02053 chr3:180459321_+GTATAC
chr12:90422241_+TGATACGGCGACCACC
chr2:46878474_-A
chr2:46878491_+ACTA
VAV2 chr9:133792011_+CA
chr1:208691946_+TAATGATACG
ADGRL3 chr4:61475596_+CGGTGGTCGCCGTA
chr4:133371297_+TCGGTGGT
chr2:185270681_+CGGTGGTCGCCGTATC
OPCML chr11:132991934_+GTCGCCGTATCA
AC010967.1 chr2:52774498_+CACCG
AL358944.1 chr11:30294394_-AAAGCTAT
SSH2 chr17:29837613_-CT
chr2:139740045_+TACGGCGACCACCG
AL023755.1 chr1:168410460_+GGGGCTG
HMGCLL1 chr6:55481554_+TATTTTT
chr3:78236057_+TTTTG
AC022325.2 chr4:175191587_+TGGTCGCCGTA
chr18:73891793_-GAGCCA
AC010619.2;RPL13A chr19:49487437_C>T
CDCA7L chr7:21924443_+TACGGCGACC
LINC00276 chr2:13978980_+TGATACGGC
chr2:123448175_+CGGTGGTCG
HSD17B4 chr5:119481623_+CGGTGG
50-75
25-50
10-25
5-10
1-5
0-1
SNVs
Insertions
Deletions
Coding
Non-coding
Ig/TCR
Repeat overlap
b
SNVs
MNVs
Insertions
Deletions
Coding
Non-coding
IG/TCR
samples
200
100
10
2
LINC02431
AC106744.1
FAM149A
ROBO1
LINC01208
ADGRG6
B4GALT1
IG/TCR
IG/TCR
HEPH
chr1
chr2
chr3
chr4
chr5
chr6
chr7
chr8
chr9
chr10
chr11
chr12
chr13
chr14
chr15
chr16
chr17
chr18
chr19
chr20
chr21
chr22
chrX
chrY

Appendix Figure S2. Top recurrent hotspots and their distribution across the genome. a) Heatmap showing mutational frequencies of the top recurrent hotspots bearing 60 or more mutated samples. Rows show hotspots (and genomic elements overlapping them, if any) found by HotspotFinder. Hotspots are denoted by their genomic coordinates, and mutation alternate. Columns show cancer types (sample size in parenthesis). The number of mutated samples and the corresponding mutational frequency in the cancer type are shown in each square. Leftmost columns represent annotations of the mutation type, region annotation class, and overlap of a repetitive sequence of hotspots as identified by HotspotFinder. **b**) Distribution of hotspots for each mutation type across the mappable genome. Hotspot shape illustrates whether the hotspot overlaps coding, non-coding or immunoglobulin and T-cell receptor regions. Regions of 10 or more consecutive low mappable megabases are shown in grey.

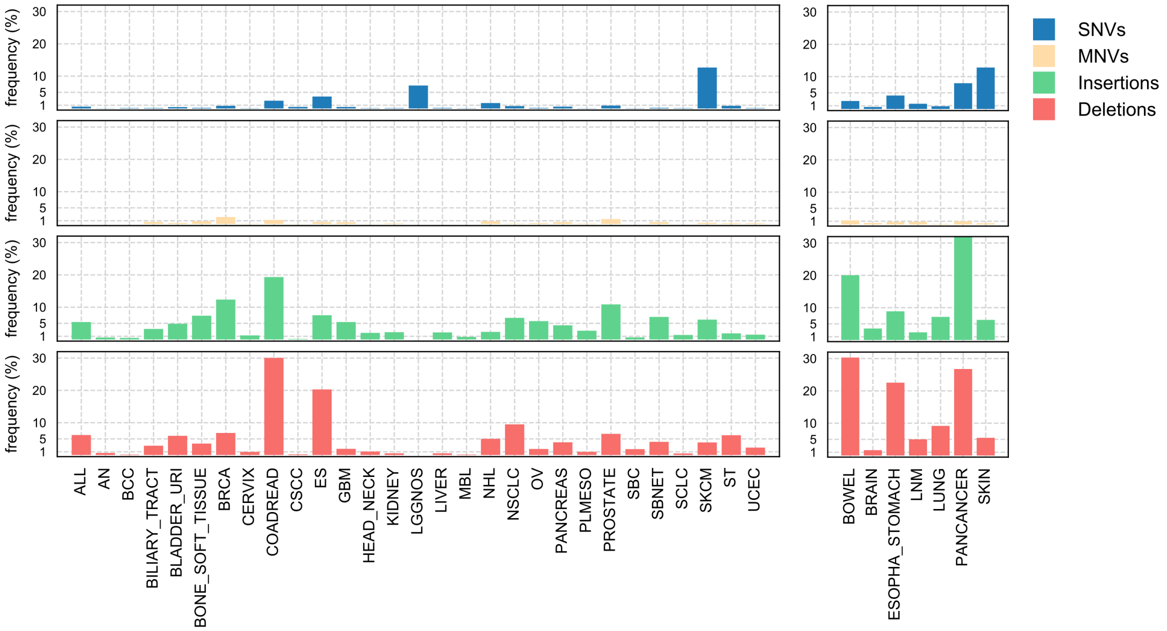

Appendix Figure S3. Frequency of hotspots formation across mutation types. Bars showing the percentage of mutations that overlap a hotspot across cancers and mutation types.

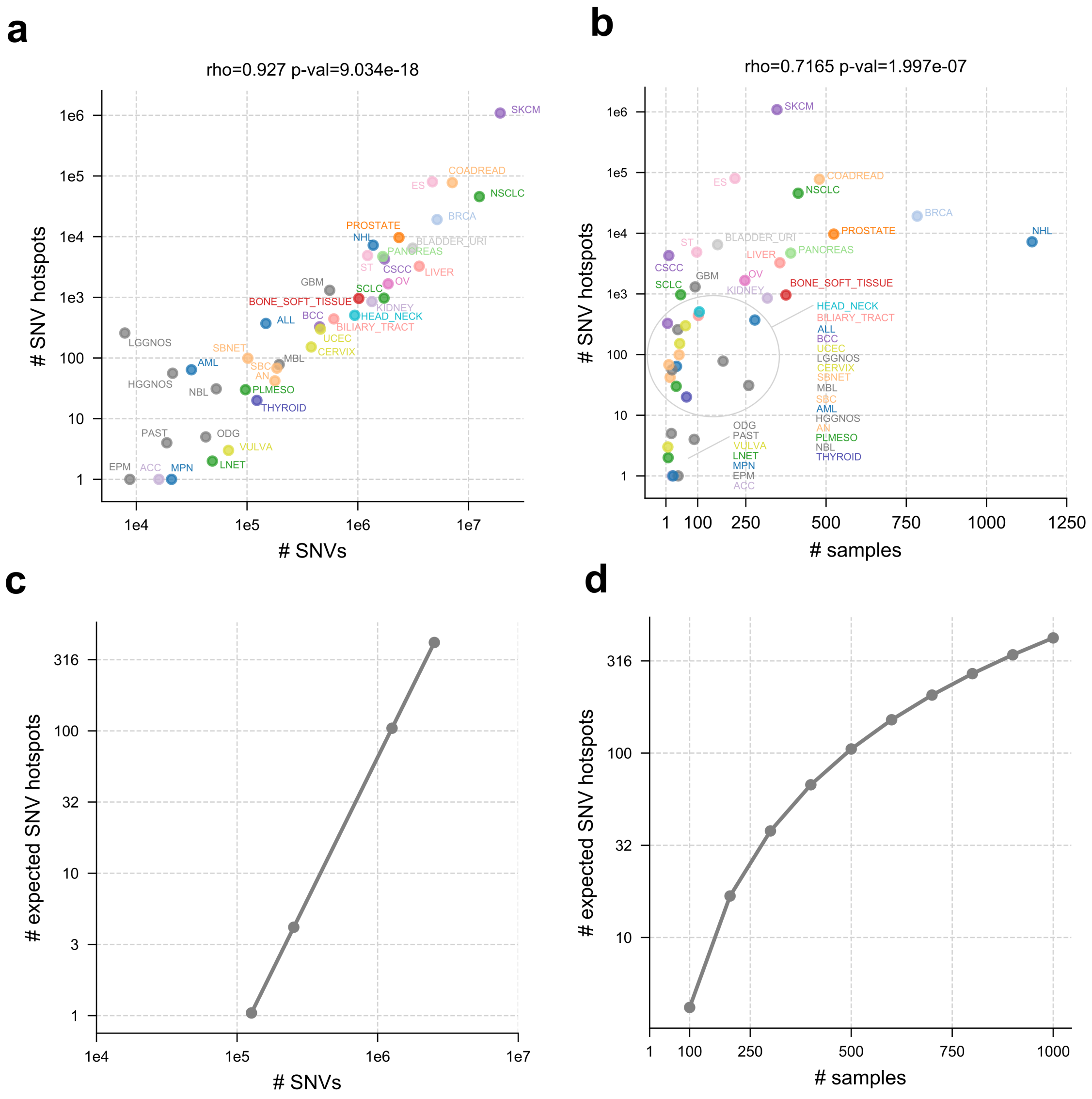

Appendix Figure S4. Relationship between the increase of hotspot burden, mutation burden and sample size. a) Relationship between number of observed hotspots and mutations across cancer types. Spearman's rho correlation coefficient among both variables and resulting p-value is shown on top. **b**) Correlation among observed SNV hotspots and sample size across cancer types. Spearman's rho correlation coefficient among both variables and resulting p-value is shown on top. **c**) Expected number of hotspots generated under a theoretical model using 100 samples with equal mutation burden and homogeneous distribution of trinucleotide-specific mutation rates across the genome (Appendix Note 5). **d**) Expected number of hotspots under the same theoretical model shown in c across different sample sizes with equal mutation burden (1 SNV / Mbp per sample).

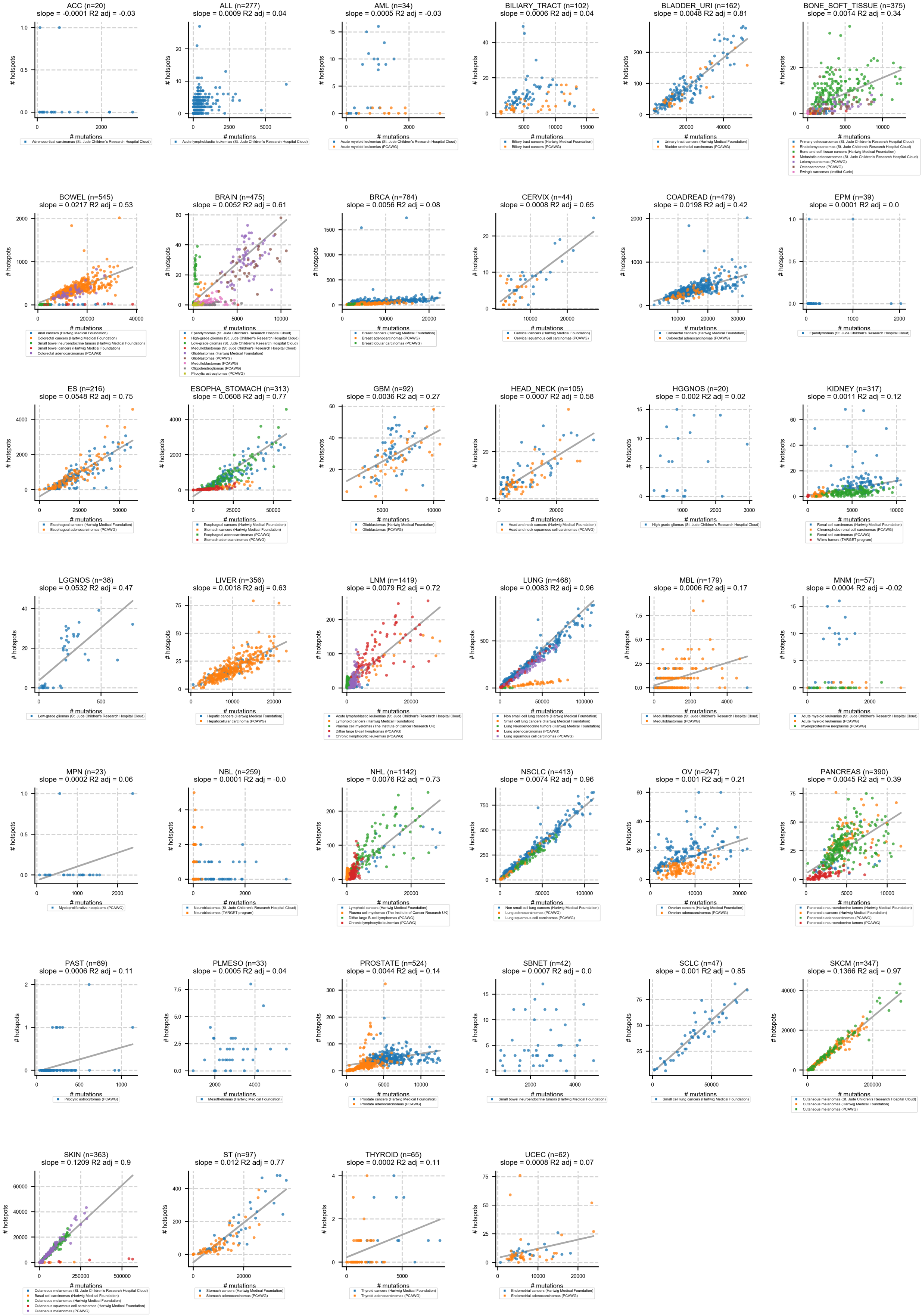
ACC (n=20)
slope = -0.0001 R2 adj = -0.03
ALL (n=277)
slope = 0.0009 R2 adj = 0.04
AML (n=34)
slope = 0.0005 R2 adj = -0.03
BILIARY_TRACT (n=102)
slope = 0.0006 R2 adj = 0.04
BLADDER_URI (n=162)
slope = 0.0048 R2 adj = 0.81
BONE_SOFT_TISSUE (n=375)
slope = 0.0014 R2 adj = 0.34
BOWEL (n=545)
slope = 0.0217 R2 adj = 0.53
BRAIN (n=475)
slope = 0.0052 R2 adj = 0.61
BRCA (n=784)
slope = 0.0056 R2 adj = 0.08
CERVIX (n=44)
slope = 0.0008 R2 adj = 0.65
COADREAD (n=479)
slope = 0.0198 R2 adj = 0.42
EPM (n=39)
slope = 0.0001 R2 adj = 0.0
ES (n=216)
slope = 0.0548 R2 adj = 0.75
ESOPHA_STOMACH (n=313)
slope = 0.0608 R2 adj = 0.77
GBM (n=92)
slope = 0.0036 R2 adj = 0.27
HEAD_NECK (n=105)
slope = 0.0007 R2 adj = 0.58
HGGNOS (n=20)
slope = 0.002 R2 adj = 0.02
KIDNEY (n=317)
slope = 0.0011 R2 adj = 0.12
LGGNOS (n=38)
slope = 0.0532 R2 adj = 0.47
LIVER (n=356)
slope = 0.0018 R2 adj = 0.63
LNM (n=1419)
slope = 0.0079 R2 adj = 0.72
LUNG (n=468)
slope = 0.0083 R2 adj = 0.96
MBL (n=179)
slope = 0.0006 R2 adj = 0.17
MNM (n=57)
slope = 0.0004 R2 adj = -0.02
MPN (n=23)
slope = 0.0002 R2 adj = 0.06
NBL (n=259)
slope = 0.0001 R2 adj = -0.0
NHL (n=1142)
slope = 0.0076 R2 adj = 0.73
NSCLC (n=413)
slope = 0.0074 R2 adj = 0.96
OV (n=247)
slope = 0.001 R2 adj = 0.21
PANCREAS (n=390)
slope = 0.0045 R2 adj = 0.39
PAST (n=89)
slope = 0.0006 R2 adj = 0.11
PLMESO (n=33)
slope = 0.0005 R2 adj = 0.04
PROSTATE (n=524)
slope = 0.0044 R2 adj = 0.14
SBNET (n=42)
slope = 0.0007 R2 adj = 0.0
SCLC (n=47)
slope = 0.001 R2 adj = 0.85
SKCM (n=347)
slope = 0.1366 R2 adj = 0.97
SKIN (n=363)
slope = 0.1209 R2 adj = 0.9
ST (n=97)
slope = 0.012 R2 adj = 0.77
THYROID (n=65)
slope = 0.0002 R2 adj = 0.11
UCEC (n=62)
slope = 0.0008 R2 adj = 0.07
# hotspots
# mutations

Appendix Figure S5. Relationship between observed hotspots and mutations. Scatter plots showing the number of SNV hotspots and mutations per sample across cancer types. Only datasets containing SNV hotspots and 20 or more samples are plotted. Slope and adjusted R^2 from linear regression models are shown on top of each plot. Regression lines are depicted for cancer types with model adjusted R^2 greater than 0.05. Samples colouring follows the sequencing cohort of origin, shown on the legend below.

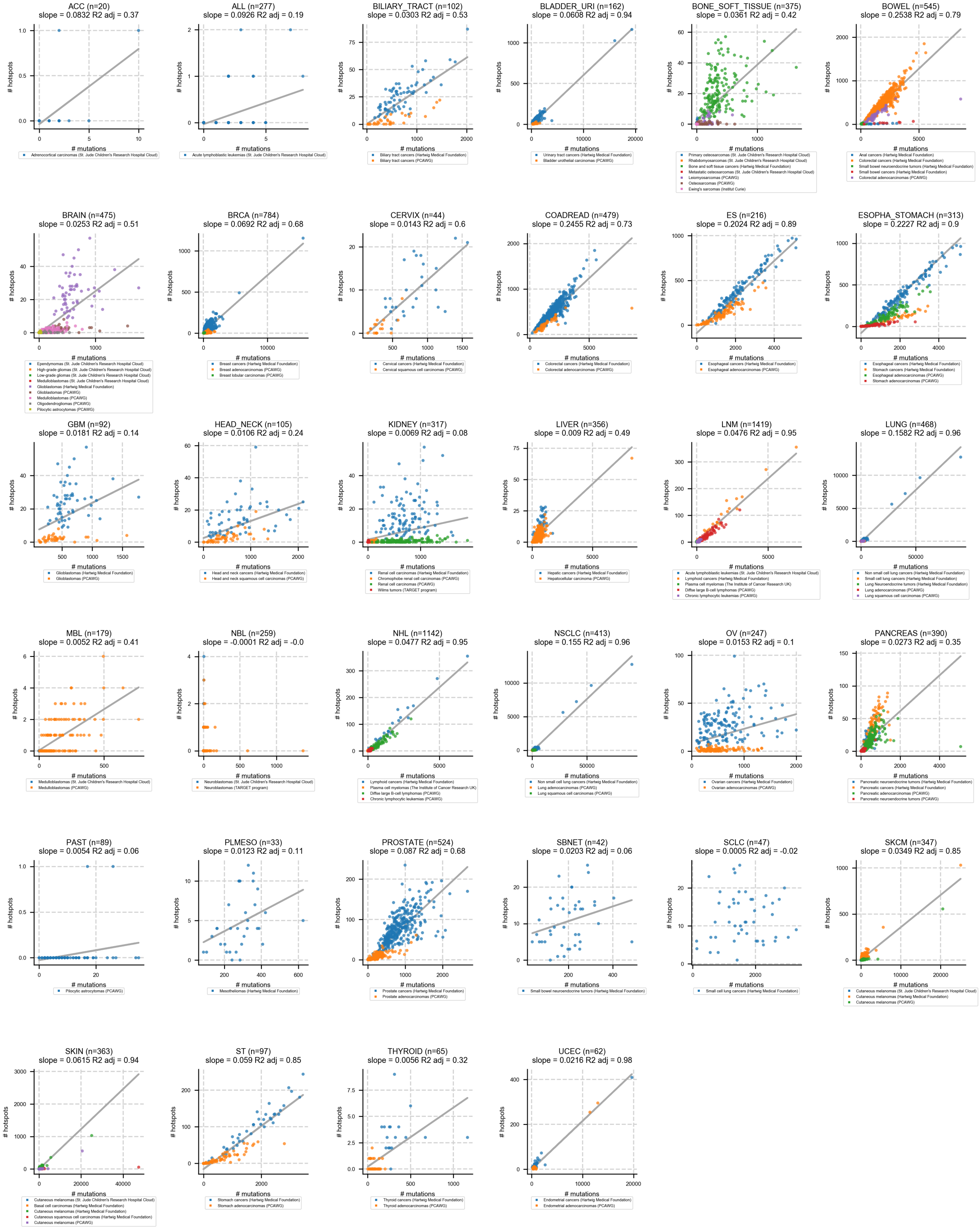
ACC (n=20)
slope = 0.0832 R2 adj = 0.37
ALL (n=277)
slope = 0.0926 R2 adj = 0.19
BILIARY_TRACT (n=102)
slope = 0.0303 R2 adj = 0.53
BLADDER_URI (n=162)
slope = 0.0608 R2 adj = 0.94
BONE_SOFT_TISSUE (n=375)
slope = 0.0361 R2 adj = 0.42
BOWEL (n=545)
slope = 0.2538 R2 adj = 0.79
BRAIN (n=475)
slope = 0.0253 R2 adj = 0.51
BRCA (n=784)
slope = 0.0692 R2 adj = 0.68
CERVIX (n=44)
slope = 0.0143 R2 adj = 0.6
COADREAD (n=479)
slope = 0.2455 R2 adj = 0.73
ES (n=216)
slope = 0.2024 R2 adj = 0.89
ESOPHA_STOMACH (n=313)
slope = 0.2227 R2 adj = 0.9
GBM (n=92)
slope = 0.0181 R2 adj = 0.14
HEAD_NECK (n=105)
slope = 0.0106 R2 adj = 0.24
KIDNEY (n=317)
slope = 0.0069 R2 adj = 0.08
LIVER (n=356)
slope = 0.009 R2 adj = 0.49
LNM (n=1419)
slope = 0.0476 R2 adj = 0.95
LUNG (n=468)
slope = 0.1582 R2 adj = 0.96
MBL (n=179)
slope = 0.0052 R2 adj = 0.41
NBL (n=259)
slope = -0.0001 R2 adj = -0.0
NHL (n=1142)
slope = 0.0477 R2 adj = 0.95
NSCLC (n=413)
slope = 0.155 R2 adj = 0.96
OV (n=247)
slope = 0.0153 R2 adj = 0.1
PANCREAS (n=390)
slope = 0.0273 R2 adj = 0.35
PAST (n=89)
slope = 0.0054 R2 adj = 0.06
PLMESO (n=33)
slope = 0.0123 R2 adj = 0.11
PROSTATE (n=524)
slope = 0.087 R2 adj = 0.68
SBNET (n=42)
slope = 0.0203 R2 adj = 0.06
SCLC (n=47)
slope = 0.0005 R2 adj = -0.02
SKCM (n=347)
slope = 0.0349 R2 adj = 0.85
SKIN (n=363)
slope = 0.0615 R2 adj = 0.94
ST (n=97)
slope = 0.059 R2 adj = 0.85
THYROID (n=65)
slope = 0.0056 R2 adj = 0.32
UCEC (n=62)
slope = 0.0216 R2 adj = 0.98
# hotspots
# mutations

Appendix Figure S6. Relationship between observed indel hotspots and mutations. Scatter plots showing the number of indel (merge of insertion and deletion) hotspots and mutations per sample across cancer types. Only datasets containing indel hotspots and 20 or more samples are plotted. Slope and adjusted R^2 from linear regression models are shown on top of each plot. Regression lines are depicted for cancer types with model adjusted R^2 greater than 0.05. Samples colouring follows the sequencing cohort of origin, shown on the legend below.

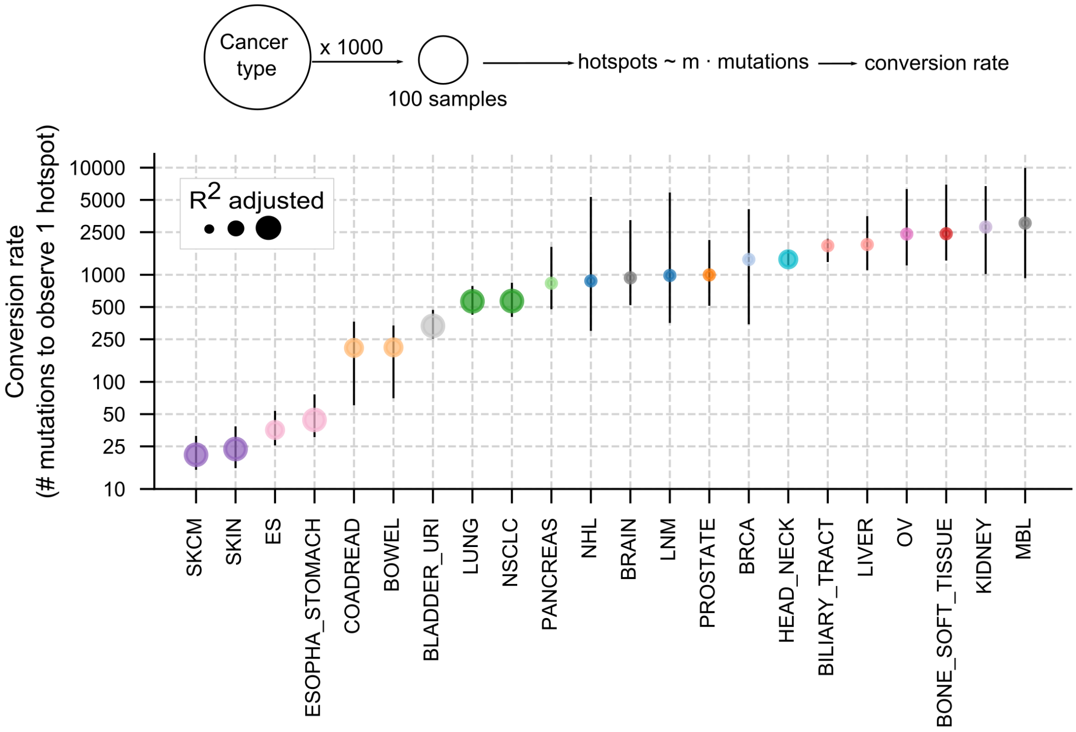

Appendix Figure S7. Conversion rates. Median conversion rates (number of mutations to observe 1 hotspot) across cancer types with more than 100 samples and at least 750 significant linear models across random replicates (mean=978.9 models per cancer type, range=777-1000; Materials and Methods). Dot size represents the goodness of fit of the linear model (R^2 adjusted). Error bars show the range of conversion rates across significant replicates.

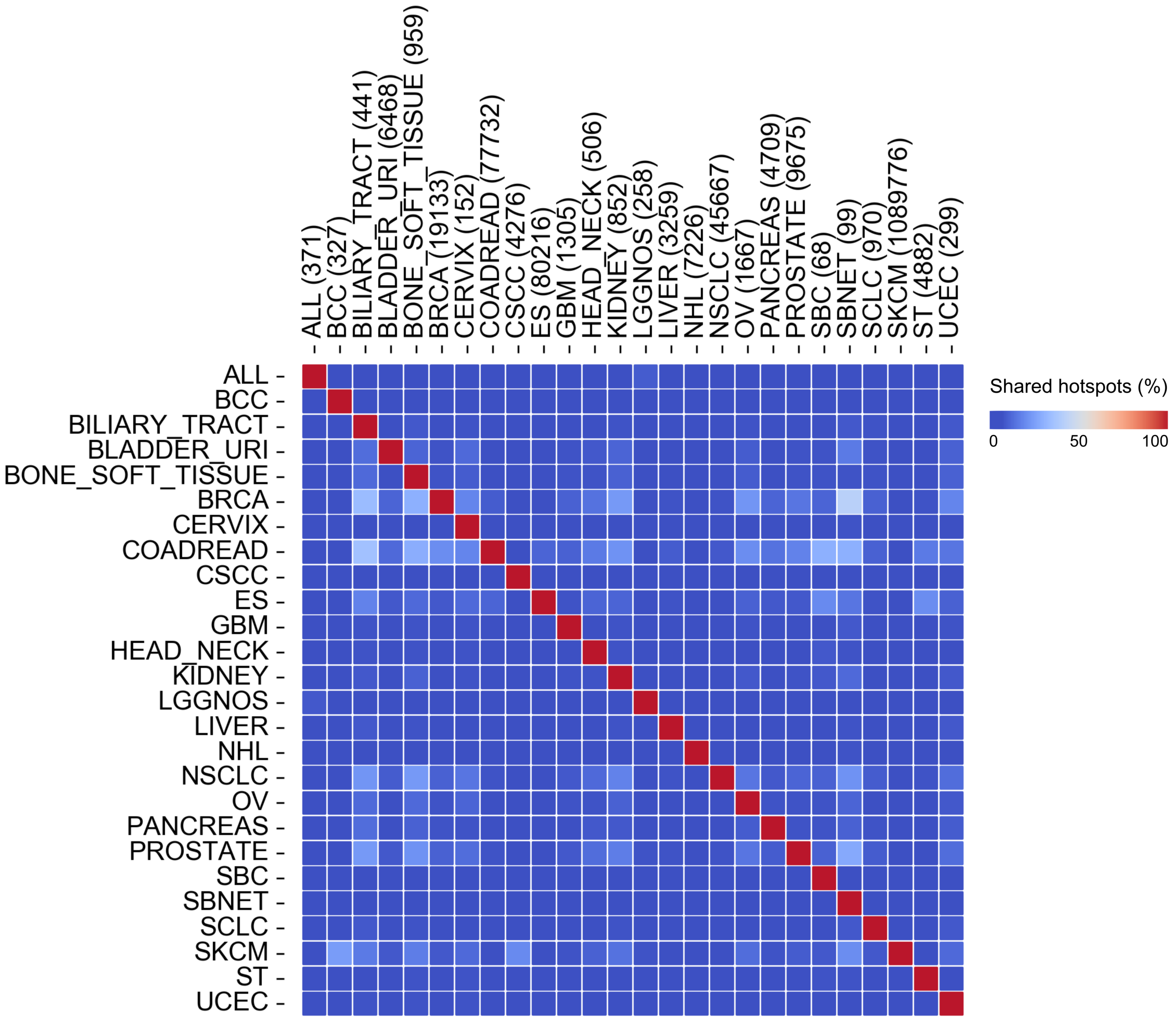

Appendix Figure S8. Hotspots shared between cancer types. Heatmap depicting the fraction of hotspots shared between cancer types with at least 100 hotspots. For each cancer type pair, the colour of the box depicts the fraction of hotspots shared with respect to the cancer type shown in the columns. The number of hotspots per cancer type is shown on top.

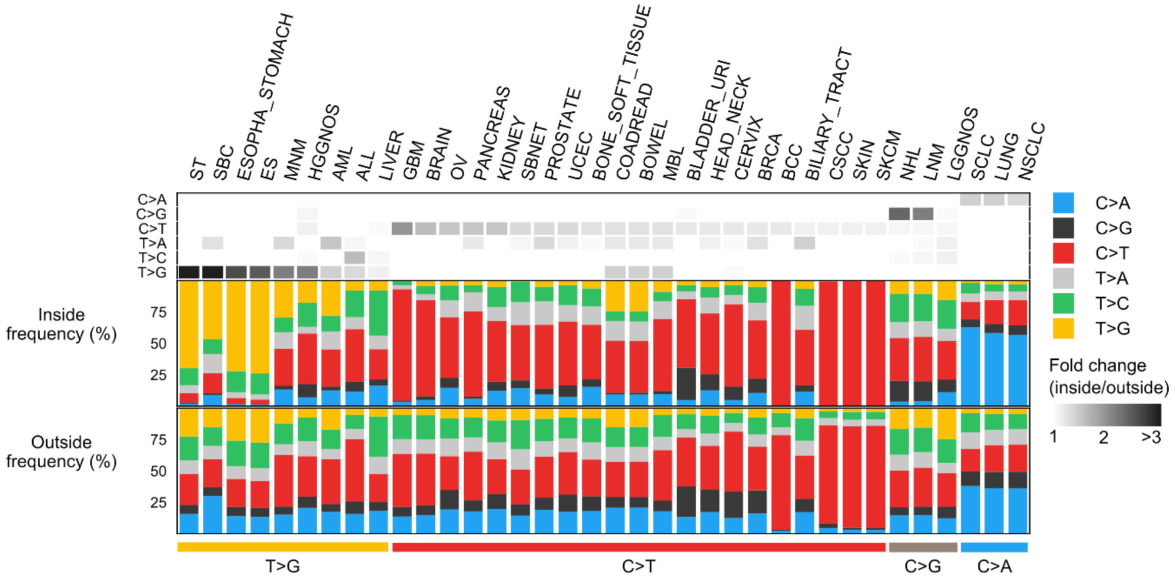

Appendix Figure S9. Hotspot enrichment across six pyrimidine-based substitution types. Enrichments (fold change > 1) of different substitution types across mutations inside hotspots versus outside hotspots across cancer types bearing 100 or more hotspot mutations. The frequency of each substitution type inside and outside hotspots are shown at the bottom panels. Cancer types appear clustered through hierarchical clustering based on their fold-changes across substitution types. Cancer types within clusters are sorted following the descending magnitude of fold change.

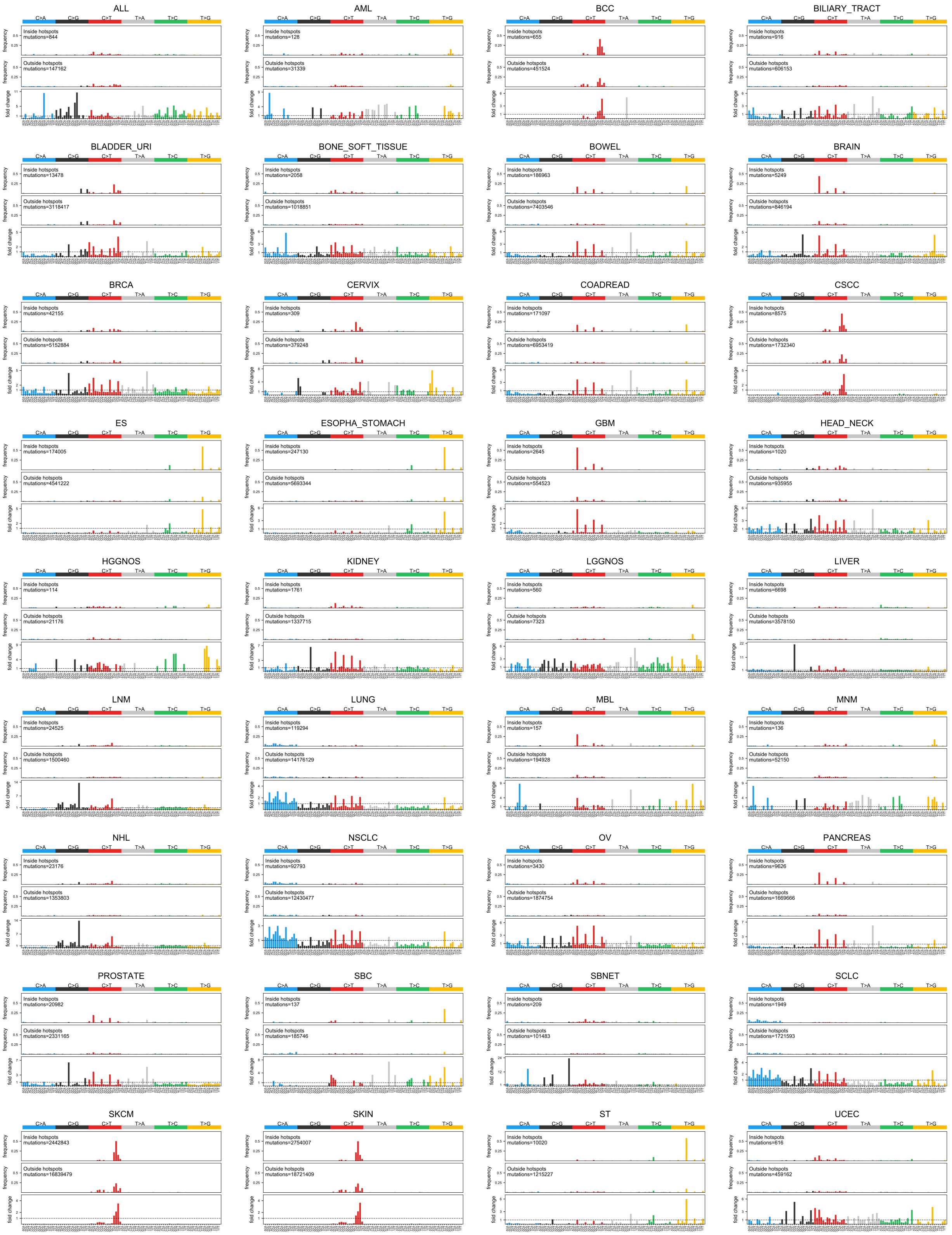
C>A C>G C>T T>A T>C T>G
frequency
fold change
ALL
Inside hotspots mutations=844
Outside hotspots mutations=147162
AML
Inside hotspots mutations=128
Outside hotspots mutations=31339
BCC
Inside hotspots mutations=655
Outside hotspots mutations=451524
BILIARY_TRACT
Inside hotspots mutations=916
Outside hotspots mutations=606153
BLADDER_URI
Inside hotspots mutations=13478
Outside hotspots mutations=3118417
BONE_SOFT_TISSUE
Inside hotspots mutations=2058
Outside hotspots mutations=1018851
BOWEL
Inside hotspots mutations=186963
Outside hotspots mutations=7403546
BRAIN
Inside hotspots mutations=5249
Outside hotspots mutations=846194
BRCA
Inside hotspots mutations=42155
Outside hotspots mutations=5152884
CERVIX
Inside hotspots mutations=309
Outside hotspots mutations=379248
COADREAD
Inside hotspots mutations=171097
Outside hotspots mutations=6953419
CSCC
Inside hotspots mutations=8575
Outside hotspots mutations=1732340
ES
Inside hotspots mutations=174005
Outside hotspots mutations=4541222
ESOPHA_STOMACH
Inside hotspots mutations=247130
Outside hotspots mutations=5693344
GBM
Inside hotspots mutations=2645
Outside hotspots mutations=554523
HEAD_NECK
Inside hotspots mutations=1020
Outside hotspots mutations=935955
HGGNOS
Inside hotspots mutations=114
Outside hotspots mutations=21176
KIDNEY
Inside hotspots mutations=1761
Outside hotspots mutations=1337715
LGGNOS
Inside hotspots mutations=560
Outside hotspots mutations=7323
LIVER
Inside hotspots mutations=6698
Outside hotspots mutations=3578150
LNM
Inside hotspots mutations=24525
Outside hotspots mutations=1500460
LUNG
Inside hotspots mutations=119294
Outside hotspots mutations=14176129
MBL
Inside hotspots mutations=157
Outside hotspots mutations=194928
MNM
Inside hotspots mutations=136
Outside hotspots mutations=52150
NHL
Inside hotspots mutations=23176
Outside hotspots mutations=1353803
NSCLC
Inside hotspots mutations=92793
Outside hotspots mutations=12430477
OV
Inside hotspots mutations=3430
Outside hotspots mutations=1874754
PANCREAS
Inside hotspots mutations=9626
Outside hotspots mutations=1669666
PROSTATE
Inside hotspots mutations=20982
Outside hotspots mutations=2331165
SBC
Inside hotspots mutations=137
Outside hotspots mutations=185746
SBNET
Inside hotspots mutations=209
Outside hotspots mutations=101483
SCLC
Inside hotspots mutations=1949
Outside hotspots mutations=1721593
SKCM
Inside hotspots mutations=2442843
Outside hotspots mutations=16839479
SKIN
Inside hotspots mutations=2754007
Outside hotspots mutations=18721409
ST
Inside hotspots mutations=10020
Outside hotspots mutations=1215227
UCEC
Inside hotspots mutations=616
Outside hotspots mutations=459162

Appendix Figure S10. Hotspot enrichment across 96-based trinucleotide single base substitutions. Frequencies of hotspot (top) versus non-hotspot (middle) mutations across the 96 types of trinucleotide based substitutions (pyrimidine-based substitutions considering all possible combinations of nucleotides immediately upstream and downstream the mutated base). Inside-to-outside fold changes across trinucleotides are shown at the bottom panel of each plot. Fold changes above 1 (dashed grey line) denote an increase of mutations inside hotspots over the background (outside) mutation rate of the particular trinucleotide. Only cancer types with at least 100 hotspot mutations are shown.

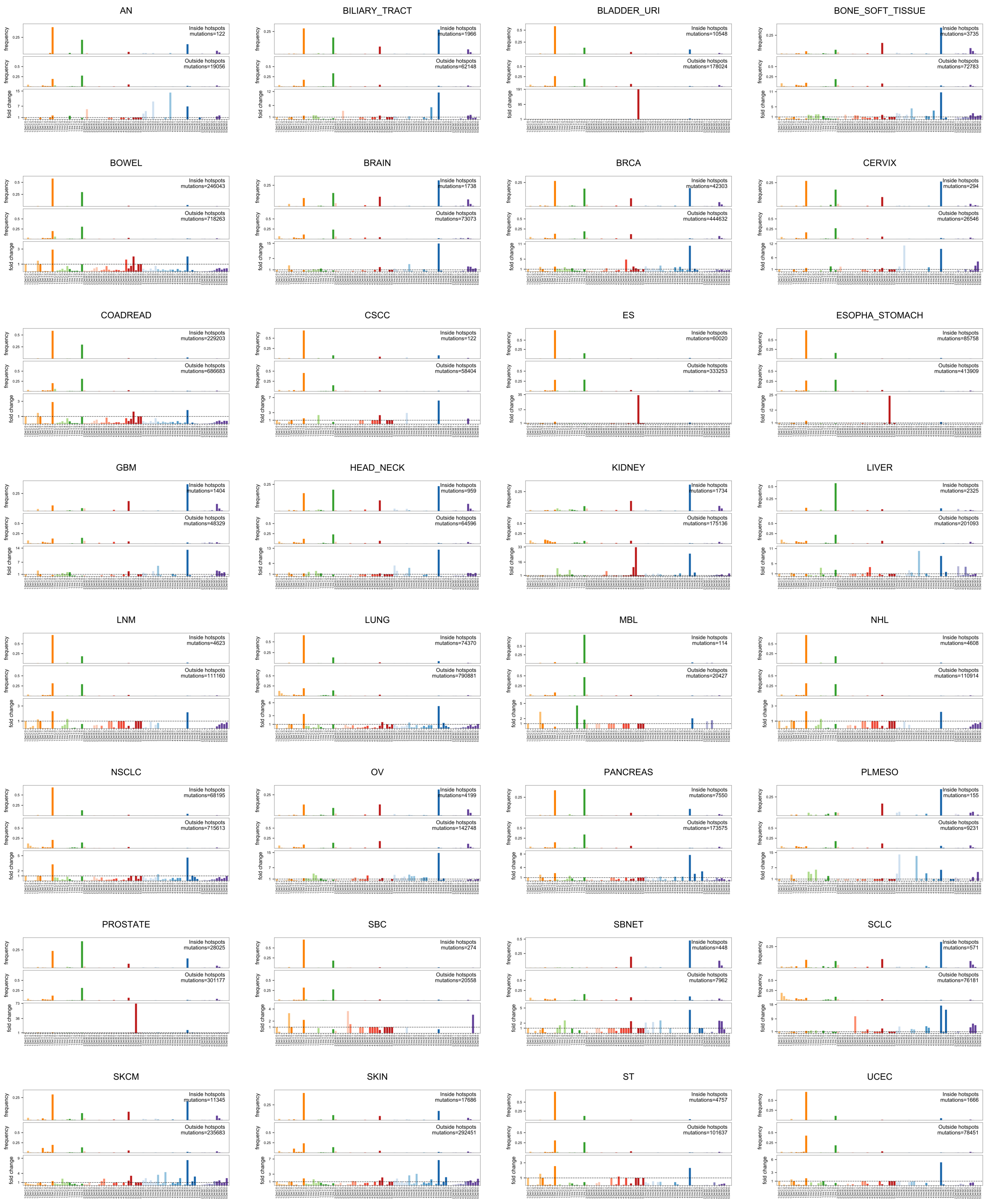
AN
Inside hotspots mutations=122
Outside hotspots mutations=19056
BILIARY_TRACT
Inside hotspots mutations=1966
Outside hotspots mutations=62148
BLADDER_URI
Inside hotspots mutations=10548
Outside hotspots mutations=178024
BONE_SOFT_TISSUE
Inside hotspots mutations=3735
Outside hotspots mutations=72783
BOWEL
Inside hotspots mutations=246043
Outside hotspots mutations=718263
BRAIN
Inside hotspots mutations=1738
Outside hotspots mutations=73073
BRCA
Inside hotspots mutations=42303
Outside hotspots mutations=444632
CERVIX
Inside hotspots mutations=294
Outside hotspots mutations=26546
COADREAD
Inside hotspots mutations=229203
Outside hotspots mutations=686683
CSCC
Inside hotspots mutations=122
Outside hotspots mutations=58404
ES
Inside hotspots mutations=60020
Outside hotspots mutations=333253
ESOPHA_STOMACH
Inside hotspots mutations=85758
Outside hotspots mutations=413909
GBM
Inside hotspots mutations=1404
Outside hotspots mutations=48329
HEAD_NECK
Inside hotspots mutations=959
Outside hotspots mutations=64596
KIDNEY
Inside hotspots mutations=1734
Outside hotspots mutations=175136
LIVER
Inside hotspots mutations=2325
Outside hotspots mutations=201093
LNM
Inside hotspots mutations=4623
Outside hotspots mutations=111160
LUNG
Inside hotspots mutations=74370
Outside hotspots mutations=790881
MBL
Inside hotspots mutations=114
Outside hotspots mutations=20427
NHL
Inside hotspots mutations=4608
Outside hotspots mutations=110914
NSCLC
Inside hotspots mutations=68195
Outside hotspots mutations=715613
OV
Inside hotspots mutations=4199
Outside hotspots mutations=142748
PANCREAS
Inside hotspots mutations=7550
Outside hotspots mutations=173575
PLMESO
Inside hotspots mutations=155
Outside hotspots mutations=9231
PROSTATE
Inside hotspots mutations=28025
Outside hotspots mutations=301177
SBC
Inside hotspots mutations=274
Outside hotspots mutations=20558
SBNET
Inside hotspots mutations=448
Outside hotspots mutations=7962
SCLC
Inside hotspots mutations=571
Outside hotspots mutations=76181
SKCM
Inside hotspots mutations=11345
Outside hotspots mutations=235683
SKIN
Inside hotspots mutations=17686
Outside hotspots mutations=292451
ST
Inside hotspots mutations=4757
Outside hotspots mutations=101637
UCEC
Inside hotspots mutations=1666
Outside hotspots mutations=78451
frequency
fold change

Appendix Figure S11. Hotspot enrichment across indel categories. Frequencies of hotspot (top) versus non-hotspot (middle) mutations across the 83-based indel categories. Inside-to-outside fold changes across each class are shown at the bottom panel of each plot. Fold changes above 1 (dashed grey line) denote an increase of mutations inside hotspots over the background (outside) mutation rate of the particular trinucleotide. Only cancer types with at least 100 indel hotspot mutations are shown.

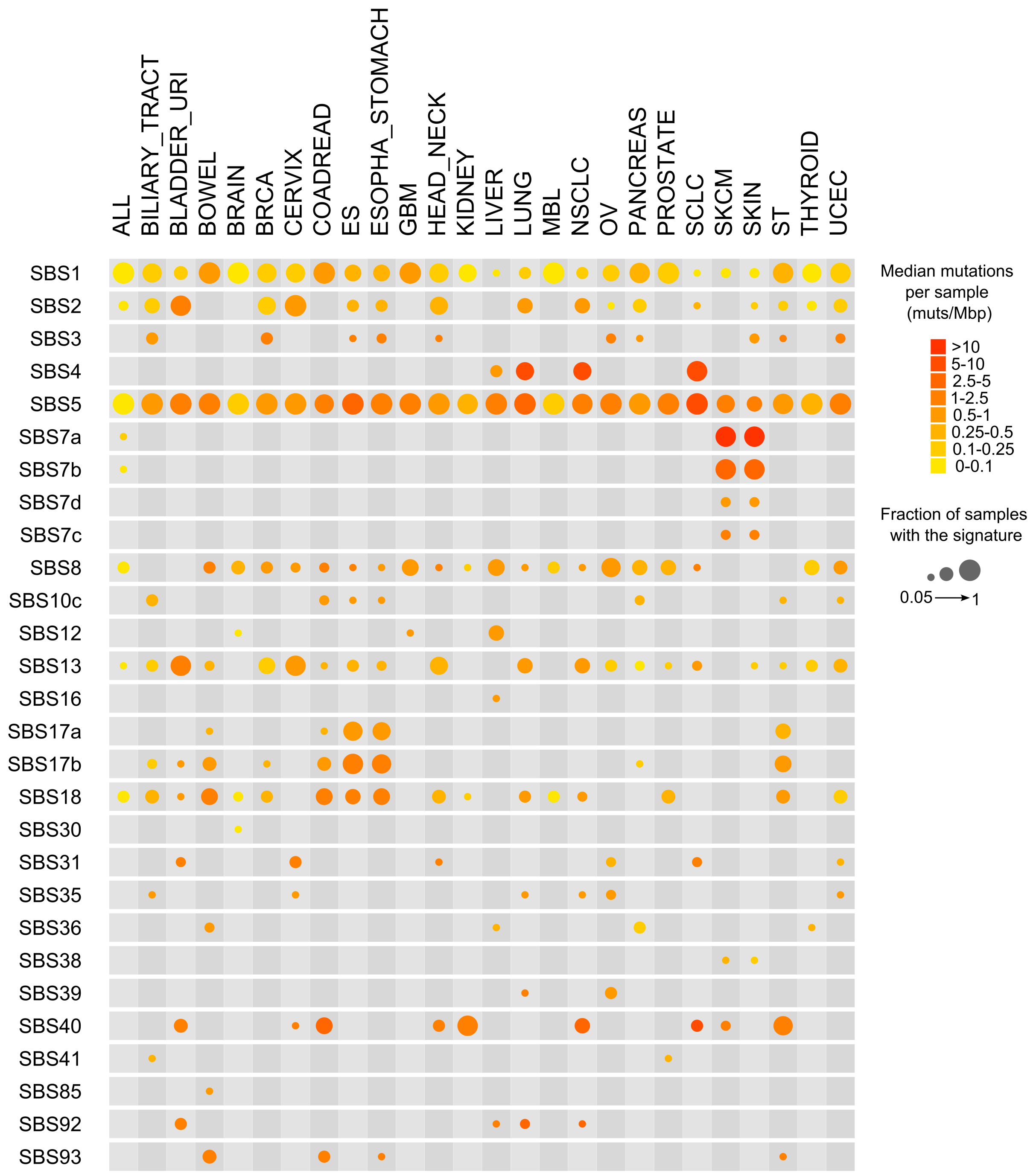

Appendix Figure S12. COSMIC reference signatures set decomposition of extracted SBS signatures. Heatmap showing the decomposition of *de novo* extracted signatures into the COSMIC v3.2 reference set. Dots show the fraction of samples of the cancer type in which the signature is found to be active. Colour shows the median mutation burden per signature across samples where the signature is active (this is, 5% or more mutations within the sample are attributable to the signature). Only signatures active in 5% or more of the samples in at least one cancer type are shown.

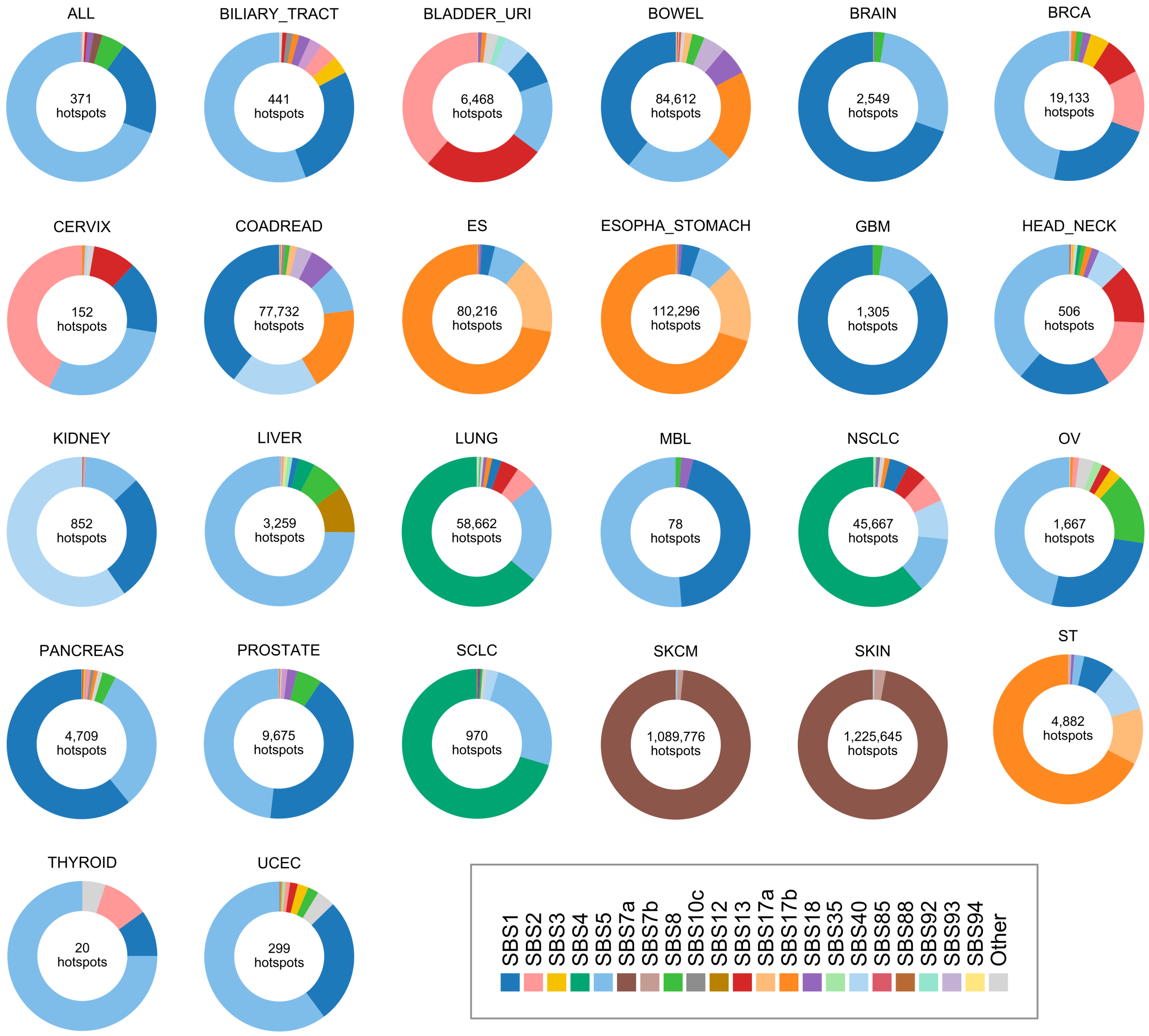

Appendix Figure S13. Proportion of hotspots per signature across cancers. Pie charts showing the proportion of hotspots attributable to signatures active in each cancer type (Materials and Methods).

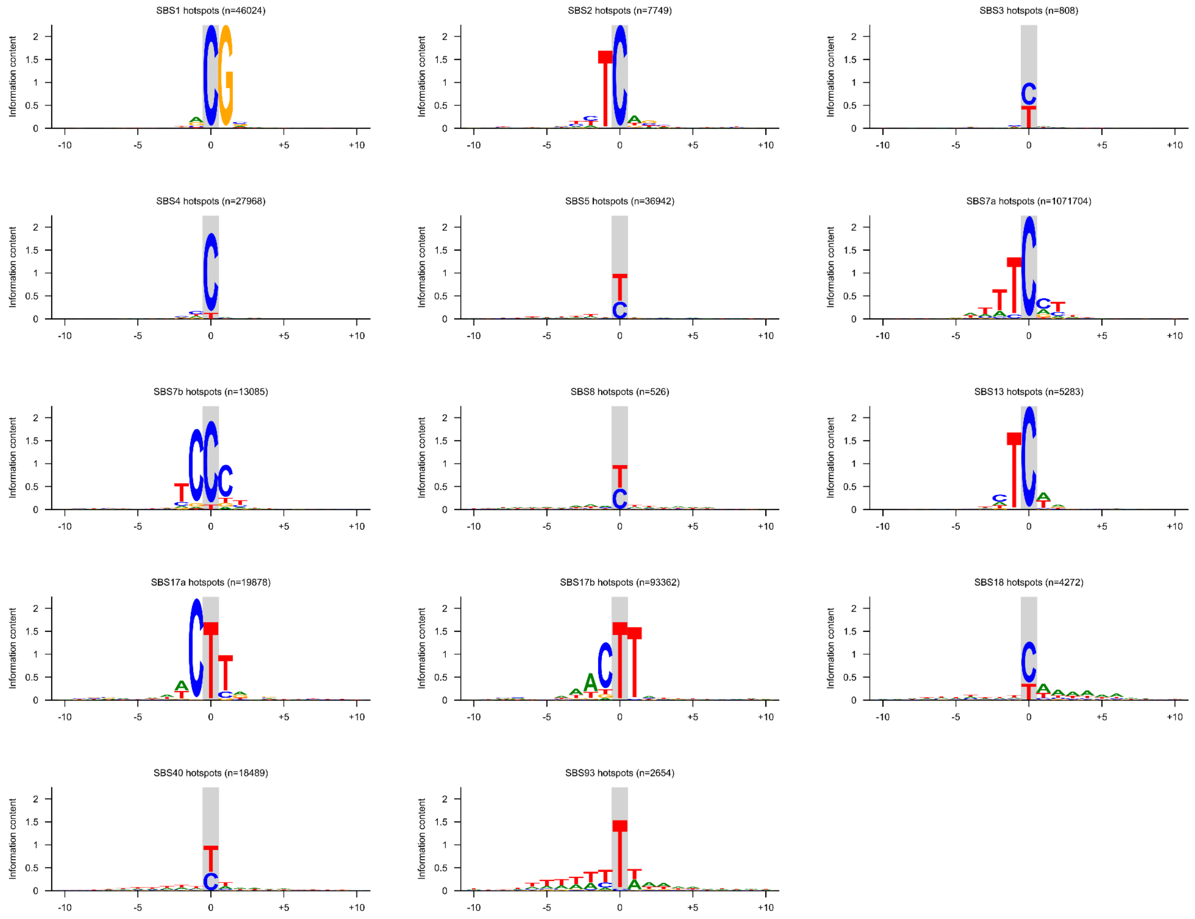

Appendix Figure S14. Hotspot logos across signatures. Logos depicting the information content of the normalised nucleotide frequency per position in a 21 bp window centred at the nucleotide with the hotspot (grey vertical line). For each signature, hotspots were merged across cancer types and their number is shown on top (Materials and Methods).

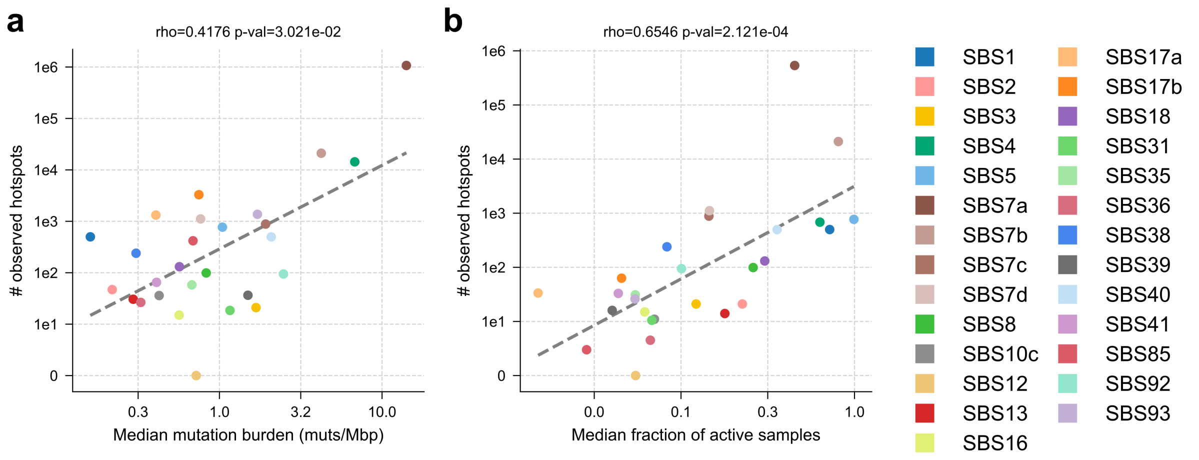

Appendix Figure S15. Relationship between hotspot burden, mutation burden and active samples per signature. a) Number of observed hotspots versus the median number of mutations attributable to the signature across cancer types where the signature is active (Materials and Methods). **b**) Number of observed hotspots versus the median fraction of active samples across cancer types where the signature is active (Materials and Methods). Dashed lines show the fitting of a linear regression (OLS) model on the data. Spearman's correlation coefficients (rho) and p-values are shown on top.

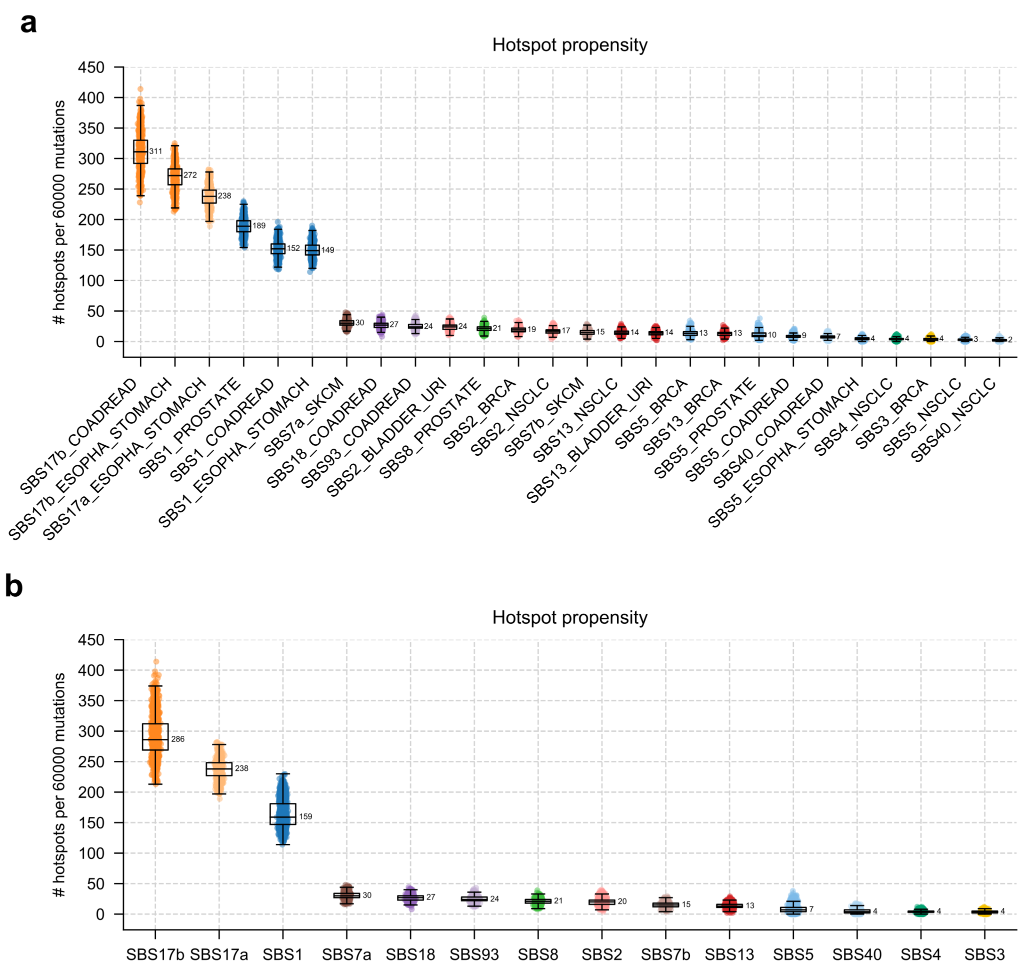

Appendix Figure S16. Hotspot propensity with increased mutation burden. a) Number of hotspots per signature and cancer type observed by subsampling 60,000 total mutations (600 mutations/sample, 100 samples). **b**) Number of hotspots per signature observed within 60,000 subsampled mutations (600 mutations/sample, 100 samples) across cancer types merging data shown in a. Error bars in a and b show 1.5 times the IQR below and above 1st and 3rd quartiles, respectively. Signatures are sorted in descending order according to the mean number of observed hotspots. Note that the number of cancer types in this experiment differs from that shown in Fig 2C-F, where we used a subsampling of 30,000 total mutations (300 mutations/sample, 100 samples). Specifically, the following signature-cancer type pairs could not be analysed do to insufficient sampling size: SBS1-BLADDER_URI, SBS1-BRCA, SBS1-NSCLC, SBS5-BLADDER_URI, and SBS17a-COADREAD.

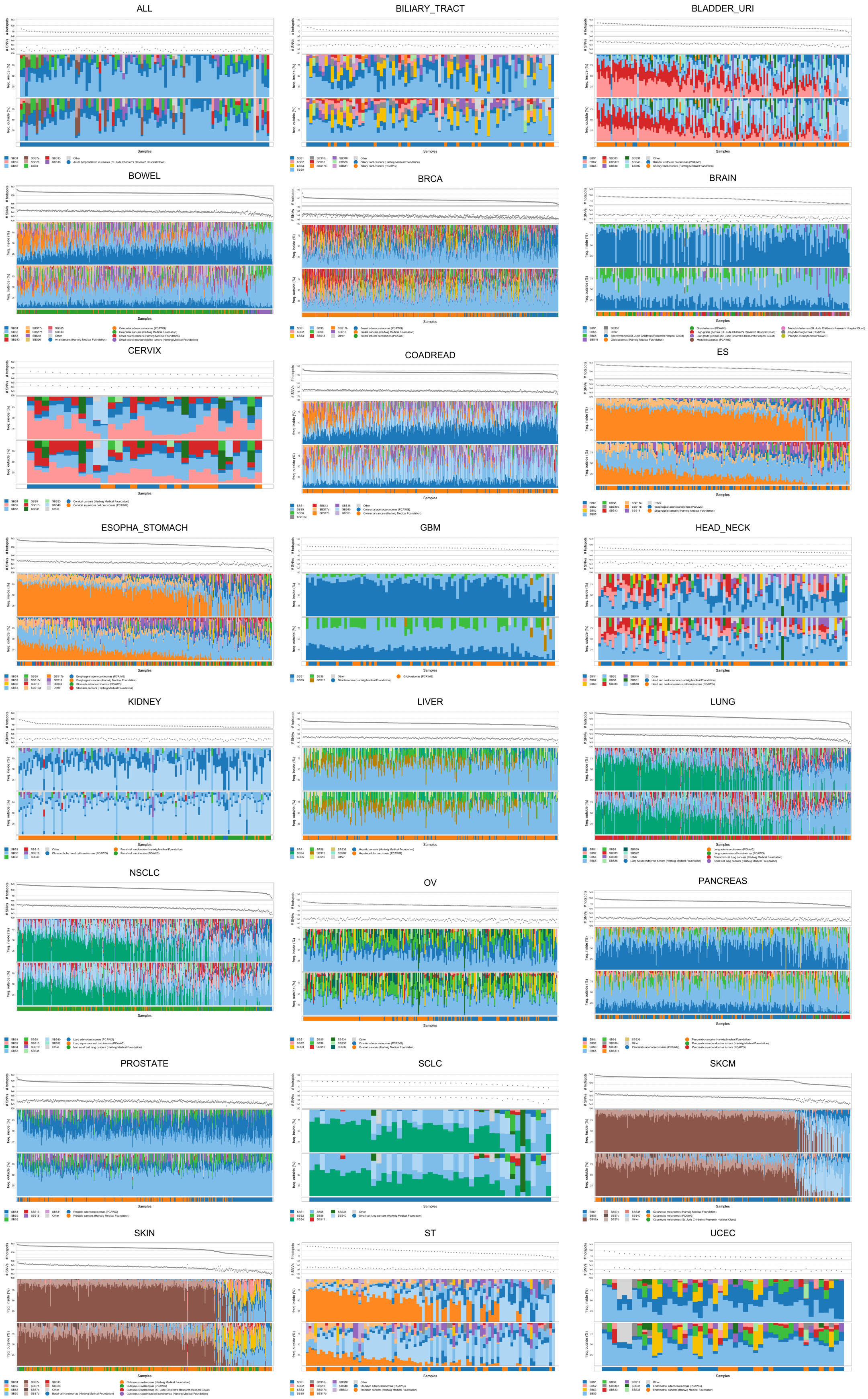
ALL
BILIARY_TRACT
BLADDER_URI
BOWEL
BRCA
BRAIN
CERVIX
COADREAD
ES
ESOPHA_STOMACH
GBM
HEAD_NECK
KIDNEY
LIVER
LUNG
NSCLC
OV
PANCREAS
PROSTATE
SCLC
SKCM
SKIN
ST
UCEC

Appendix Figure S17. Signature frequency inside and outside hotspots. Barplots of the relative frequency of mutational signatures per sample within mutations inside (top) and outside hotspots (bottom) per cancer type. Signatures that are active in the cancer type (those that are present in 5% or more samples with a frequency larger than 5% of mutations per sample) are coloured following each legend, or labelled as “Other”. Only samples with 3 or more hotspots are shown. MBL and THYROID cancer types, among which only 8 and 2 samples have 3 or more hotspots, respectively, are not included.

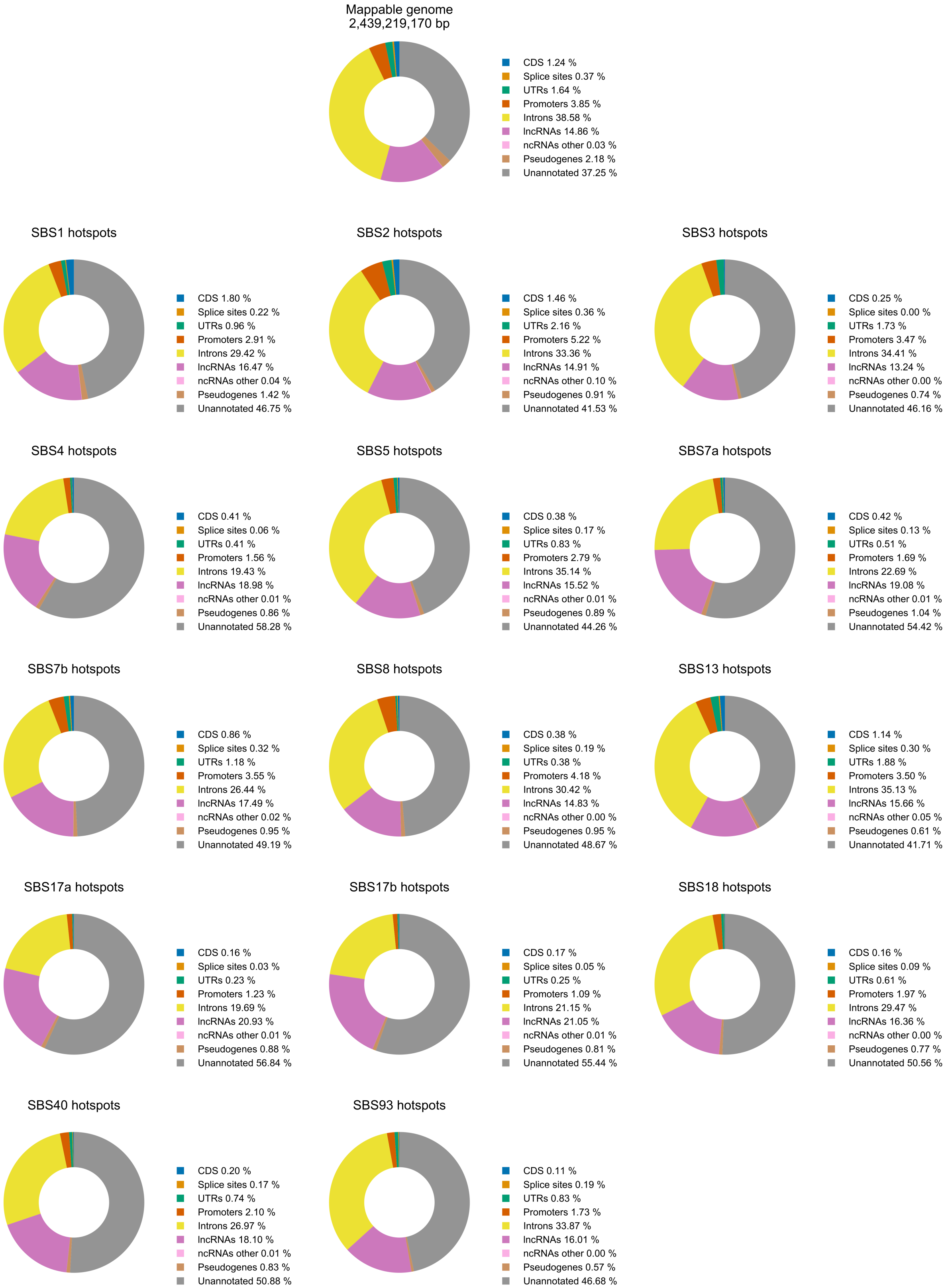
Mappable genome
2,439,219,170 bp
CDS 1.24 %
Splice sites 0.37 %
UTRs 1.64 %
Promoters 3.85 %
Introns 38.58 %
lncRNAs 14.86 %
ncRNAs other 0.03 %
Pseudogenes 2.18 %
Unannotated 37.25 %
SBS1 hotspots
CDS 1.80 %
Splice sites 0.22 %
UTRs 0.96 %
Promoters 2.91 %
Introns 29.42 %
lncRNAs 16.47 %
ncRNAs other 0.04 %
Pseudogenes 1.42 %
Unannotated 46.75 %
SBS2 hotspots
CDS 1.46 %
Splice sites 0.36 %
UTRs 2.16 %
Promoters 5.22 %
Introns 33.36 %
lncRNAs 14.91 %
ncRNAs other 0.10 %
Pseudogenes 0.91 %
Unannotated 41.53 %
SBS3 hotspots
CDS 0.25 %
Splice sites 0.00 %
UTRs 1.73 %
Promoters 3.47 %
Introns 34.41 %
lncRNAs 13.24 %
ncRNAs other 0.00 %
Pseudogenes 0.74 %
Unannotated 46.16 %
SBS4 hotspots
CDS 0.41 %
Splice sites 0.06 %
UTRs 0.41 %
Promoters 1.56 %
Introns 19.43 %
lncRNAs 18.98 %
ncRNAs other 0.01 %
Pseudogenes 0.86 %
Unannotated 58.28 %
SBS5 hotspots
CDS 0.38 %
Splice sites 0.17 %
UTRs 0.83 %
Promoters 2.79 %
Introns 35.14 %
lncRNAs 15.52 %
ncRNAs other 0.01 %
Pseudogenes 0.89 %
Unannotated 44.26 %
SBS7a hotspots
CDS 0.42 %
Splice sites 0.13 %
UTRs 0.51 %
Promoters 1.69 %
Introns 22.69 %
lncRNAs 19.08 %
ncRNAs other 0.01 %
Pseudogenes 1.04 %
Unannotated 54.42 %
SBS7b hotspots
CDS 0.86 %
Splice sites 0.32 %
UTRs 1.18 %
Promoters 3.55 %
Introns 26.44 %
lncRNAs 17.49 %
ncRNAs other 0.02 %
Pseudogenes 0.95 %
Unannotated 49.19 %
SBS8 hotspots
CDS 0.38 %
Splice sites 0.19 %
UTRs 0.38 %
Promoters 4.18 %
Introns 30.42 %
lncRNAs 14.83 %
ncRNAs other 0.00 %
Pseudogenes 0.95 %
Unannotated 48.67 %
SBS13 hotspots
CDS 1.14 %
Splice sites 0.30 %
UTRs 1.88 %
Promoters 3.50 %
Introns 35.13 %
lncRNAs 15.66 %
ncRNAs other 0.05 %
Pseudogenes 0.61 %
Unannotated 41.71 %
SBS17a hotspots
CDS 0.16 %
Splice sites 0.03 %
UTRs 0.23 %
Promoters 1.23 %
Introns 19.69 %
lncRNAs 20.93 %
ncRNAs other 0.01 %
Pseudogenes 0.88 %
Unannotated 56.84 %
SBS17b hotspots
CDS 0.17 %
Splice sites 0.05 %
UTRs 0.25 %
Promoters 1.09 %
Introns 21.15 %
lncRNAs 21.05 %
ncRNAs other 0.01 %
Pseudogenes 0.81 %
Unannotated 55.44 %
SBS18 hotspots
CDS 0.16 %
Splice sites 0.09 %
UTRs 0.61 %
Promoters 1.97 %
Introns 29.47 %
lncRNAs 16.36 %
ncRNAs other 0.00 %
Pseudogenes 0.77 %
Unannotated 50.56 %
SBS40 hotspots
CDS 0.20 %
Splice sites 0.17 %
UTRs 0.74 %
Promoters 2.10 %
Introns 26.97 %
lncRNAs 18.10 %
ncRNAs other 0.01 %
Pseudogenes 0.83 %
Unannotated 50.88 %
SBS93 hotspots
CDS 0.11 %
Splice sites 0.19 %
UTRs 0.83 %
Promoters 1.73 %
Introns 33.87 %
lncRNAs 16.01 %
ncRNAs other 0.00 %
Pseudogenes 0.57 %
Unannotated 46.68 %

Appendix Figure S18. Hotspots overlapping genomic elements. Proportion of observed hotspots overlapping different categories of annotated genomic elements across signatures. The annotations consist of non-overlapping categories of genomic elements as defined in HotspotFinder (Appendix Note 1) according to the following hierarchy, where CDS has the largest priority: CDS, splice sites, UTRs, promoters, introns, and lncRNAs / other ncRNAs / pseudogenes. lncRNAs, other ncRNAs and pseudogenes do not overlap any element of the former 5 classes, however they can be overlapping among each other. Splice sites, UTRs, promoters, and introns are extracted from protein-coding genes. lncRNAs annotations include both exons, introns and promoter sequences of lncRNA genes. The proportion of the mappable genome annotated with these genomic elements is shown on top.

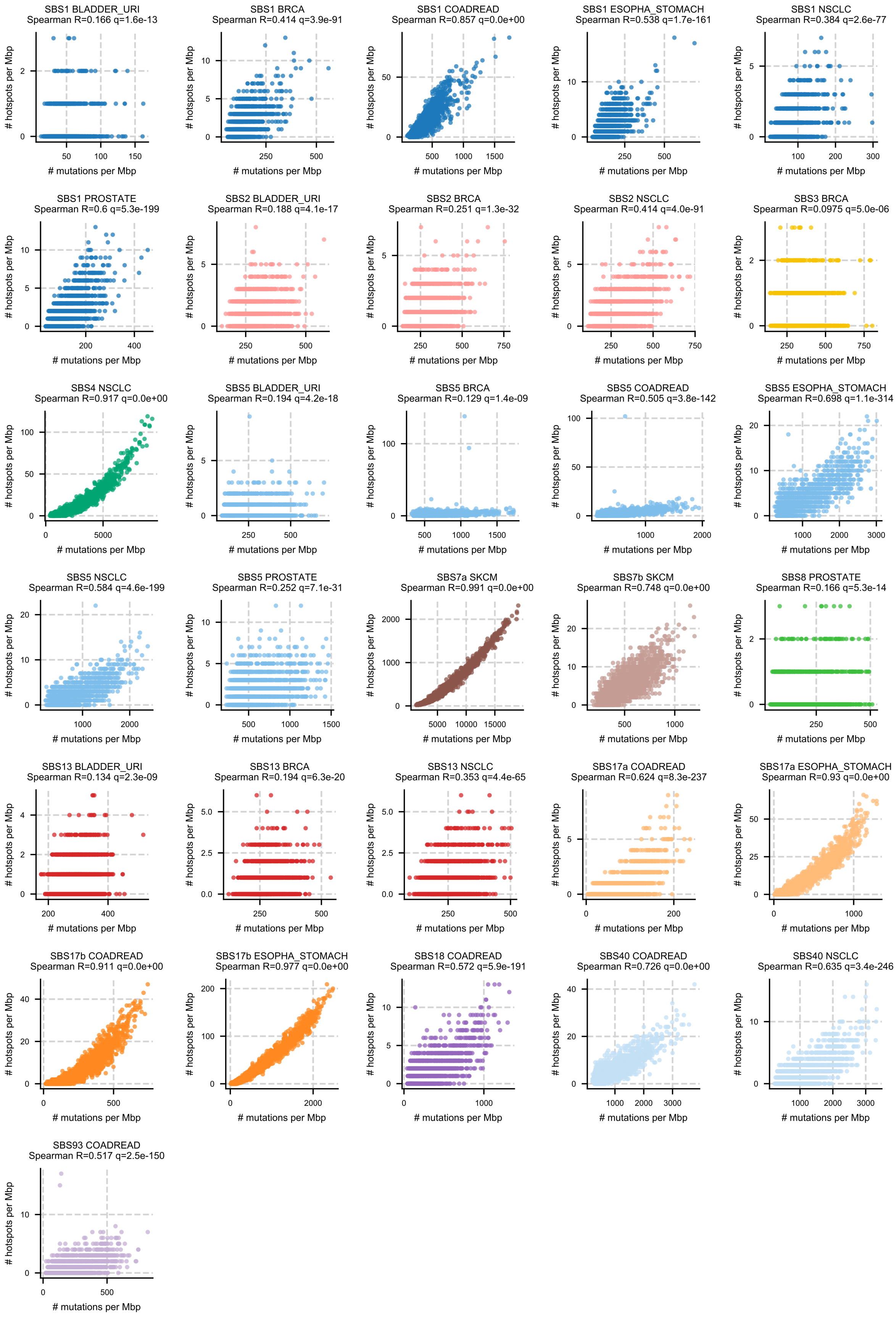
SBS1 BLADDER_URI
Spearman R=0.166 q=1.6e-13
SBS1 BRCA
Spearman R=0.414 q=3.9e-91
SBS1 COADREAD
Spearman R=0.857 q=0.0e+00
SBS1 ESOPHA_STOMACH
Spearman R=0.538 q=1.7e-161
SBS1 NSCLC
Spearman R=0.384 q=2.6e-77
SBS1 PROSTATE
Spearman R=0.6 q=5.3e-199
SBS2 BLADDER_URI
Spearman R=0.188 q=4.1e-17
SBS2 BRCA
Spearman R=0.251 q=1.3e-32
SBS2 NSCLC
Spearman R=0.414 q=4.0e-91
SBS3 BRCA
Spearman R=0.0975 q=5.0e-06
SBS4 NSCLC
Spearman R=0.917 q=0.0e+00
SBS5 BLADDER_URI
Spearman R=0.194 q=4.2e-18
SBS5 BRCA
Spearman R=0.129 q=1.4e-09
SBS5 COADREAD
Spearman R=0.505 q=3.8e-142
SBS5 ESOPHA_STOMACH
Spearman R=0.698 q=1.1e-314
SBS5 NSCLC
Spearman R=0.584 q=4.6e-199
SBS5 PROSTATE
Spearman R=0.252 q=7.1e-31
SBS7a SKCM
Spearman R=0.991 q=0.0e+00
SBS7b SKCM
Spearman R=0.748 q=0.0e+00
SBS8 PROSTATE
Spearman R=0.166 q=5.3e-14
SBS13 BLADDER_URI
Spearman R=0.134 q=2.3e-09
SBS13 BRCA
Spearman R=0.194 q=6.3e-20
SBS13 NSCLC
Spearman R=0.353 q=4.4e-65
SBS17a COADREAD
Spearman R=0.624 q=8.3e-237
SBS17a ESOPHA_STOMACH
Spearman R=0.93 q=0.0e+00
SBS17b COADREAD
Spearman R=0.911 q=0.0e+00
SBS17b ESOPHA_STOMACH
Spearman R=0.977 q=0.0e+00
SBS18 COADREAD
Spearman R=0.572 q=5.9e-191
SBS40 COADREAD
Spearman R=0.726 q=0.0e+00
SBS40 NSCLC
Spearman R=0.635 q=3.4e-246
SBS93 COADREAD
Spearman R=0.517 q=2.5e-150
hotspots per Mbp
mutations per Mbp

Appendix Figure S19. Relationship between hotspot burden and mutation burden across genome megabases. Scatter plots showing the number of hotspots and mutations attributed to a signature in a cancer type across mappable genome megabases (n = 2,196). Spearman's rho and Benjamini-Hochberg adjusted p-value of the correlation between both variables is shown on top.

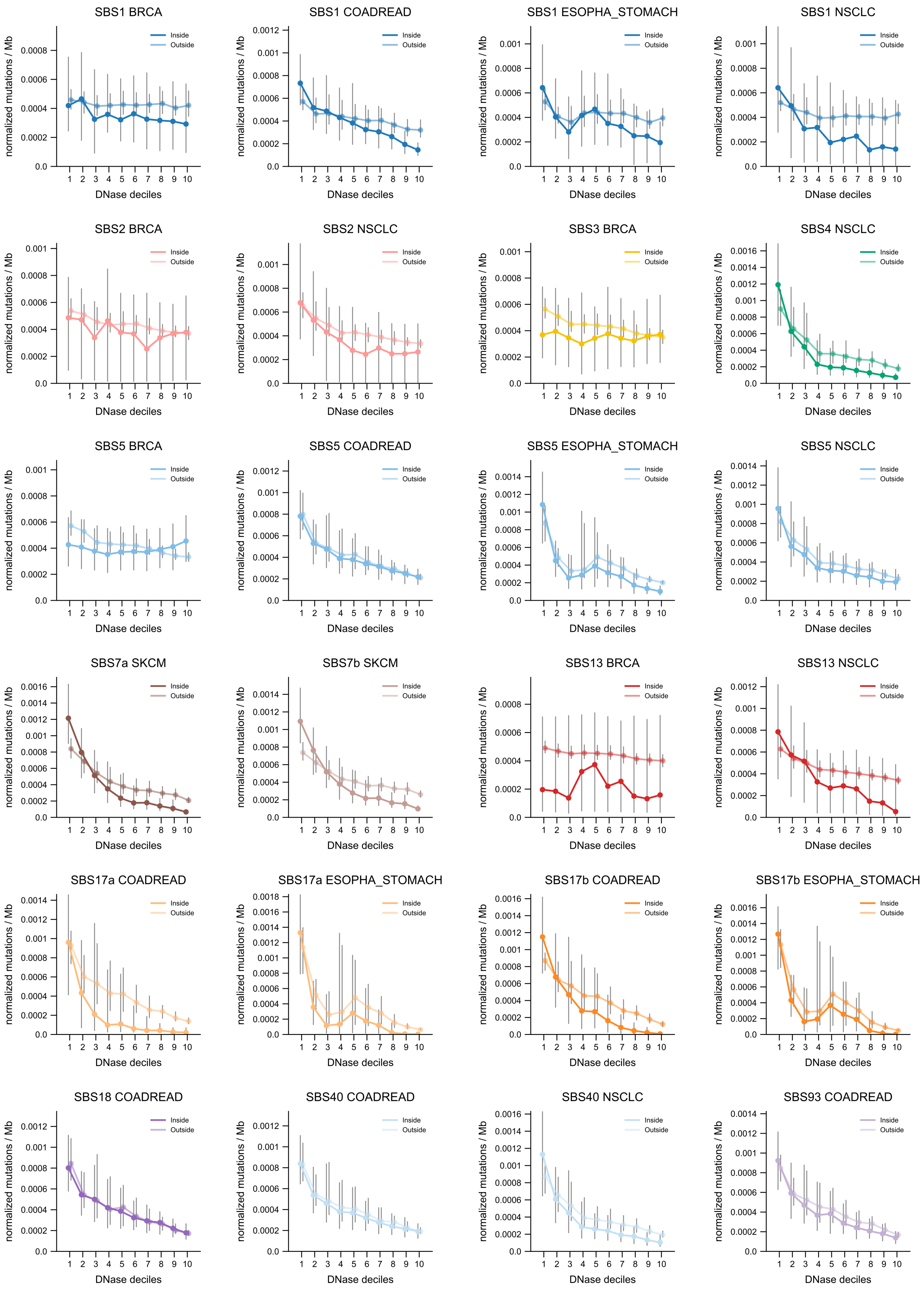
SBS1 BRCA
SBS1 COADREAD
SBS1 ESOPHA_STOMACH
SBS1 NSCLC
SBS2 BRCA
SBS2 NSCLC
SBS3 BRCA
SBS4 NSCLC
SBS5 BRCA
SBS5 COADREAD
SBS5 ESOPHA_STOMACH
SBS5 NSCLC
SBS7a SKCM
SBS7b SKCM
SBS13 BRCA
SBS13 NSCLC
SBS17a COADREAD
SBS17a ESOPHA_STOMACH
SBS17b COADREAD
SBS17b ESOPHA_STOMACH
SBS18 COADREAD
SBS40 COADREAD
SBS40 NSCLC
SBS93 COADREAD
Inside
Outside
normalized mutations / Mb
DNase deciles

Appendix Figure S20. Mutation rates across genomic regions with different chromatin accessibility. Median normalised rates of observed mutations inside and outside hotspots across mappable megabases (n = 2,196) according to DNase signal of chromatin accessibility in tissue-matched epigenomes (Materials and Methods). Bins represent deciles of accessibility (measured as relative coverage by DNAse peaks) from low (1) to high (10). Error bars show the IQR of the distribution of mutations in each decile.

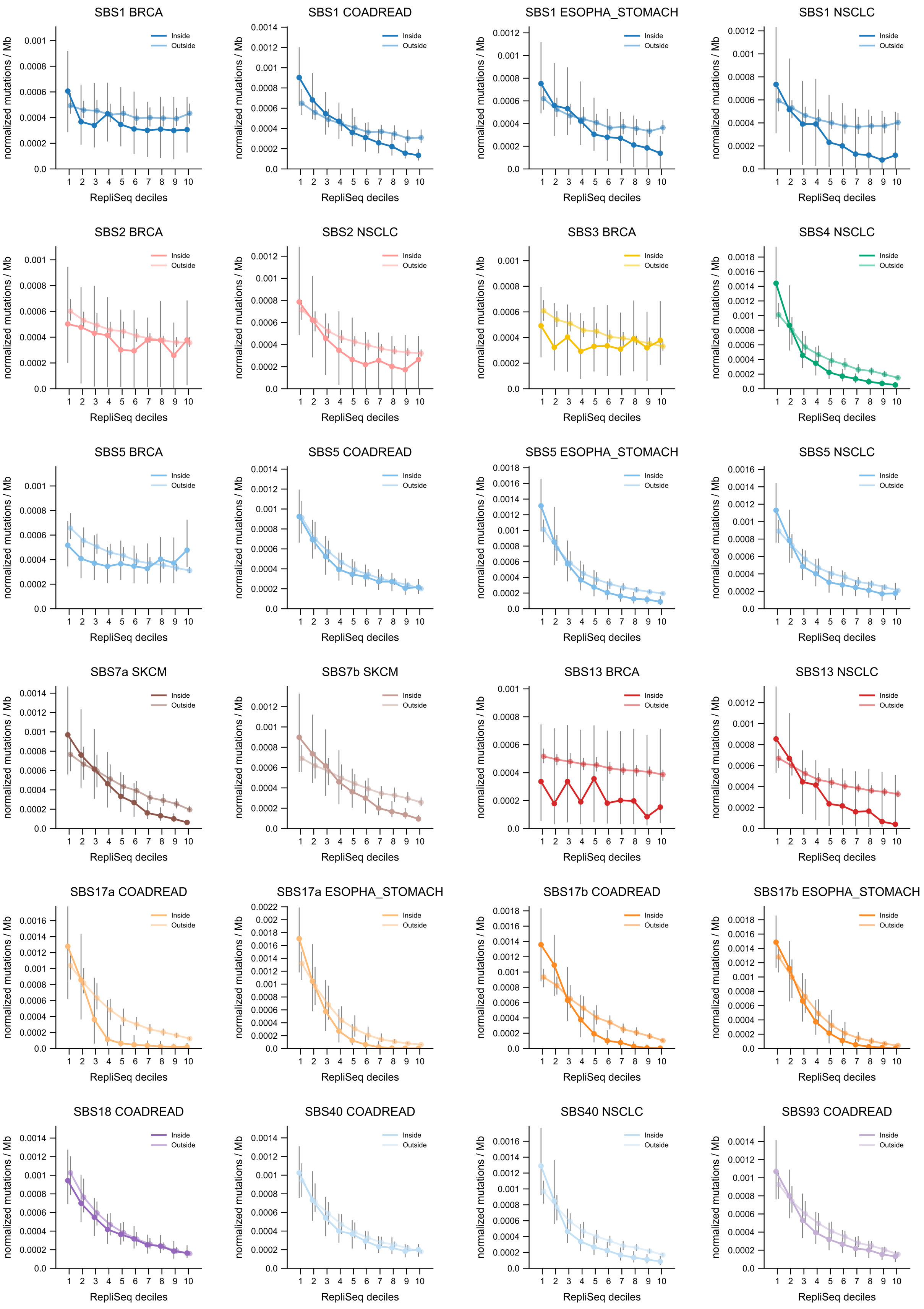
SBS1 BRCA
SBS1 COADREAD
SBS1 ESOPHA_STOMACH
SBS1 NSCLC
SBS2 BRCA
SBS2 NSCLC
SBS3 BRCA
SBS4 NSCLC
SBS5 BRCA
SBS5 COADREAD
SBS5 ESOPHA_STOMACH
SBS5 NSCLC
SBS7a SKCM
SBS7b SKCM
SBS13 BRCA
SBS13 NSCLC
SBS17a COADREAD
SBS17a ESOPHA_STOMACH
SBS17b COADREAD
SBS17b ESOPHA_STOMACH
SBS18 COADREAD
SBS40 COADREAD
SBS40 NSCLC
SBS93 COADREAD
normalized mutations / Mb
RepliSeq deciles
Inside
Outside

Appendix Figure S21. Mutation rate across genomic regions with different replication timing. Median normalised rates of observed mutations inside and outside hotspots across mappable megabases (n = 2,196) classified according to their Repli-Seq signal across 7 cell lines of solid tissues (Materials and Methods). Bins represent deciles from late (1) to early (10) replication timing. Error bars show the IQR of the distribution of mutations per decile.

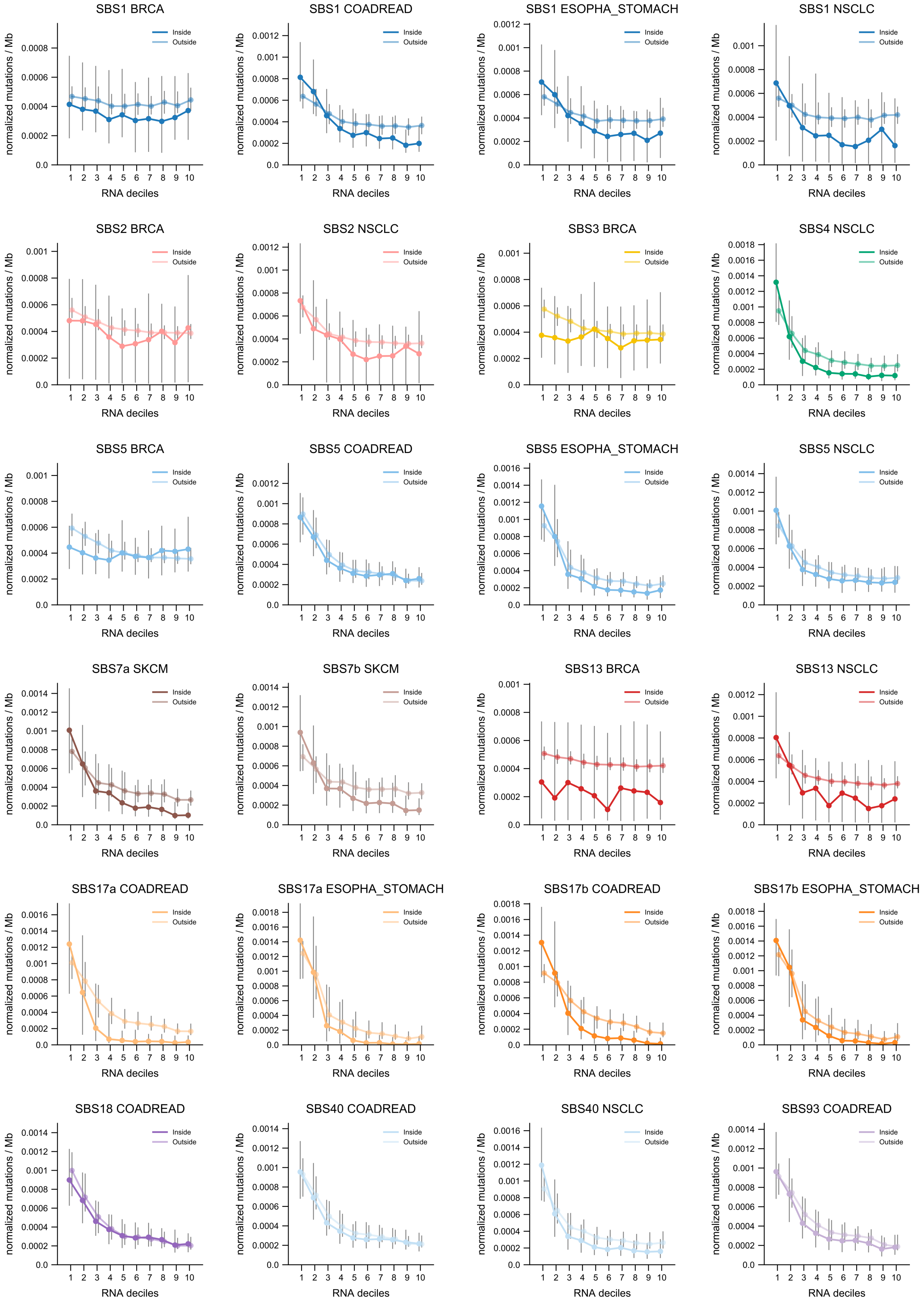
SBS1 BRCA
SBS1 COADREAD
SBS1 ESOPHA_STOMACH
SBS1 NSCLC
SBS2 BRCA
SBS2 NSCLC
SBS3 BRCA
SBS4 NSCLC
SBS5 BRCA
SBS5 COADREAD
SBS5 ESOPHA_STOMACH
SBS5 NSCLC
SBS7a SKCM
SBS7b SKCM
SBS13 BRCA
SBS13 NSCLC
SBS17a COADREAD
SBS17a ESOPHA_STOMACH
SBS17b COADREAD
SBS17b ESOPHA_STOMACH
SBS18 COADREAD
SBS40 COADREAD
SBS40 NSCLC
SBS93 COADREAD
Inside
Outside
normalized mutations / Mb
RNA deciles

Appendix Figure S22. Mutation rate across genomic regions with different transcriptional activity. Median normalised rates of observed mutations inside and outside hotspots across mappable megabases (n = 2,196) binned by RNA-Seq signal in tissue-matched epigenomes (Materials and Methods). Bins represent deciles of RNA-Seq signal from low (1) to high (10). Error bars show the IQR of the distribution of mutations per decile.

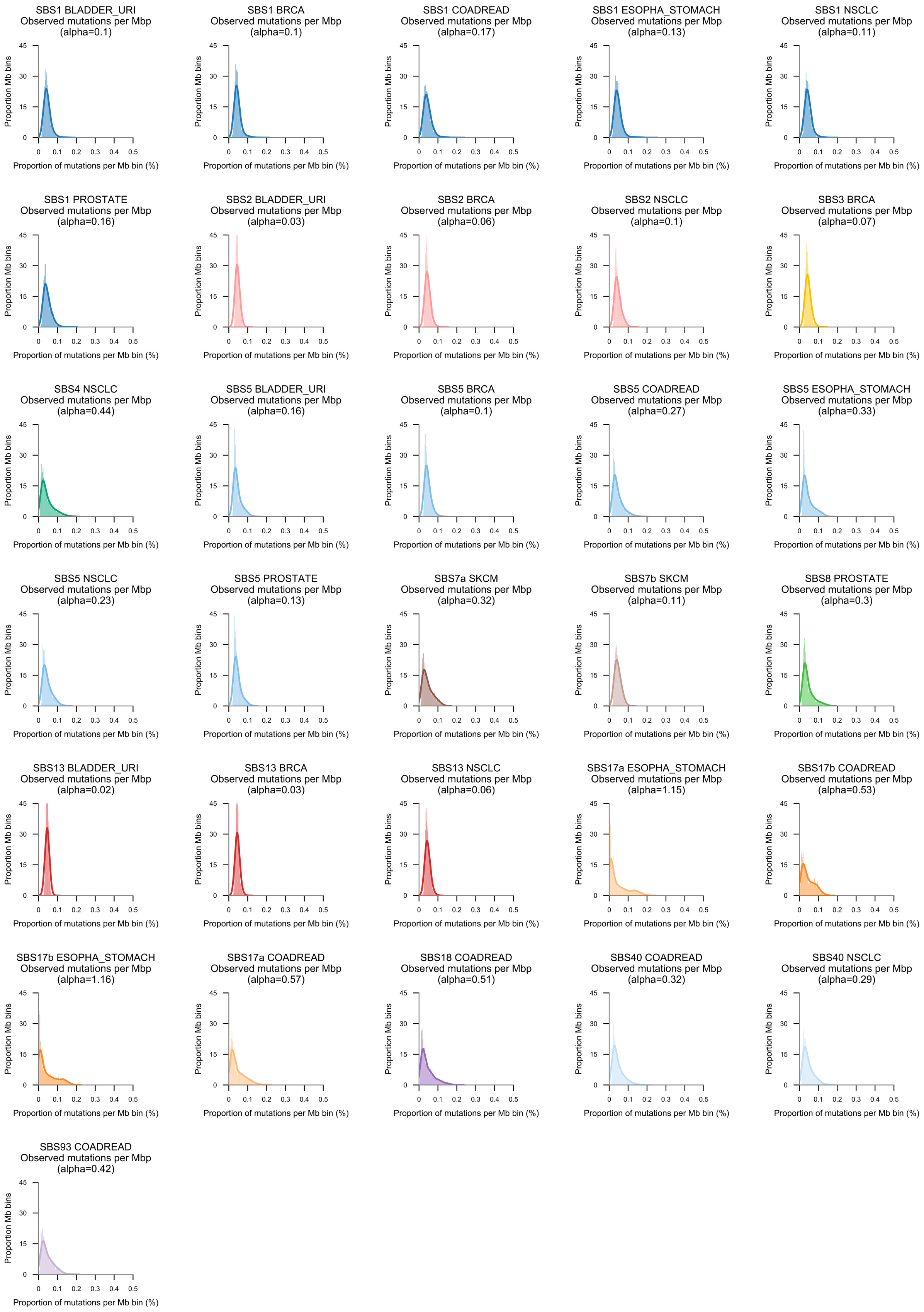
SBS1 BLADDER_URI
Observed mutations per Mbp
(alpha=0.1)
SBS1 BRCA
Observed mutations per Mbp
(alpha=0.1)
SBS1 COADREAD
Observed mutations per Mbp
(alpha=0.17)
SBS1 ESOPHA_STOMACH
Observed mutations per Mbp
(alpha=0.13)
SBS1 NSCLC
Observed mutations per Mbp
(alpha=0.11)
SBS1 PROSTATE
Observed mutations per Mbp
(alpha=0.16)
SBS2 BLADDER_URI
Observed mutations per Mbp
(alpha=0.03)
SBS2 BRCA
Observed mutations per Mbp
(alpha=0.06)
SBS2 NSCLC
Observed mutations per Mbp
(alpha=0.1)
SBS3 BRCA
Observed mutations per Mbp
(alpha=0.07)
SBS4 NSCLC
Observed mutations per Mbp
(alpha=0.44)
SBS5 BLADDER_URI
Observed mutations per Mbp
(alpha=0.16)
SBS5 BRCA
Observed mutations per Mbp
(alpha=0.1)
SBS5 COADREAD
Observed mutations per Mbp
(alpha=0.27)
SBS5 ESOPHA_STOMACH
Observed mutations per Mbp
(alpha=0.33)
SBS5 NSCLC
Observed mutations per Mbp
(alpha=0.23)
SBS5 PROSTATE
Observed mutations per Mbp
(alpha=0.13)
SBS7a SKCM
Observed mutations per Mbp
(alpha=0.32)
SBS7b SKCM
Observed mutations per Mbp
(alpha=0.11)
SBS8 PROSTATE
Observed mutations per Mbp
(alpha=0.3)
SBS13 BLADDER_URI
Observed mutations per Mbp
(alpha=0.02)
SBS13 BRCA
Observed mutations per Mbp
(alpha=0.03)
SBS13 NSCLC
Observed mutations per Mbp
(alpha=0.06)
SBS17a ESOPHA_STOMACH
Observed mutations per Mbp
(alpha=1.15)
SBS17b COADREAD
Observed mutations per Mbp
(alpha=0.53)
SBS17b ESOPHA_STOMACH
Observed mutations per Mbp
(alpha=1.16)
SBS17a COADREAD
Observed mutations per Mbp
(alpha=0.57)
SBS18 COADREAD
Observed mutations per Mbp
(alpha=0.51)
SBS40 COADREAD
Observed mutations per Mbp
(alpha=0.32)
SBS40 NSCLC
Observed mutations per Mbp
(alpha=0.29)
SBS93 COADREAD
Observed mutations per Mbp
(alpha=0.42)
Proportion Mb bins
0
15
30
45
0
0.1
0.2
0.3
0.4
0.5
Proportion of mutations per Mb bin (%)

Appendix Figure S23. Distribution of mutations across genome megabases. Histograms show the fraction of mutations across mappable genome megabases (n = 2,196). Lines represent a kernel density estimate (bandwidth=0.01) of the shape of the distribution. The overdispersion (alpha) of the underlying distribution of mutation counts across megabases is shown at the top of each plot (Materials and Methods).

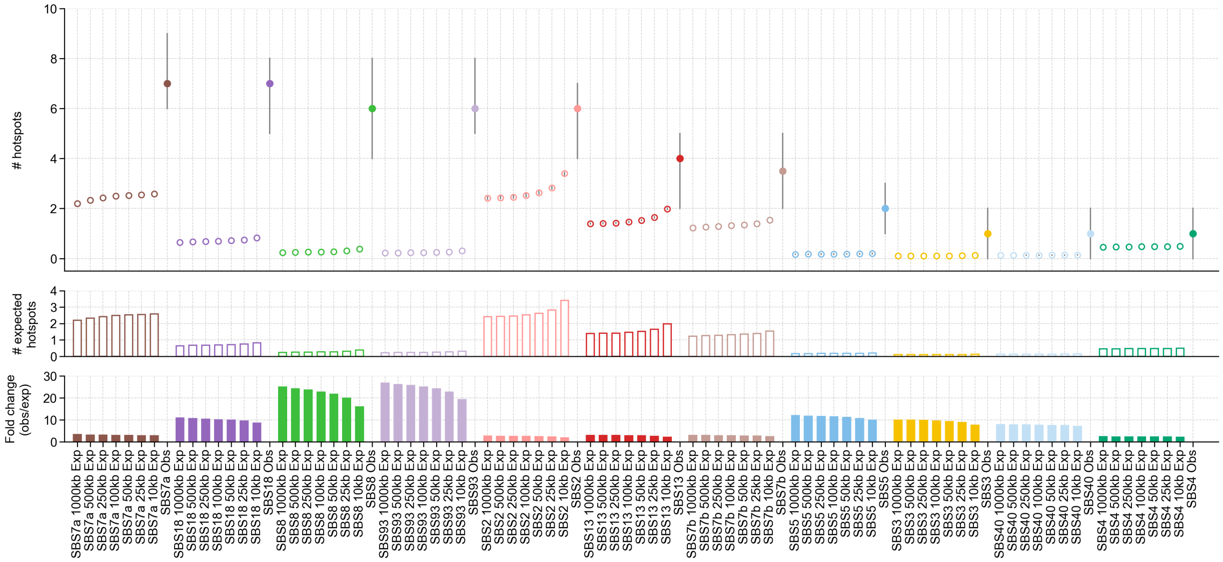

Appendix Figure S24. Comparison of observed versus expected hotspot propensity across submegabase models. Number of observed hotspots across signatures together with the expected number of hotspots computed using models accounting for trinucleotide composition and large-scale mutation rate variability for different genomic bin sizes (1 Mbp to 10 Kbp) (top). Error bars show the IQR. The number of expected hotspots per model is shown in the middle panel. The fold change of the number of observed hotspots versus expected hotspot for each model is shown in the bottom panel. Observed and expected hotspot propensities have been calculated using 30,000 mutations (300 mutations/sample and 100 samples) (Materials and Methods).

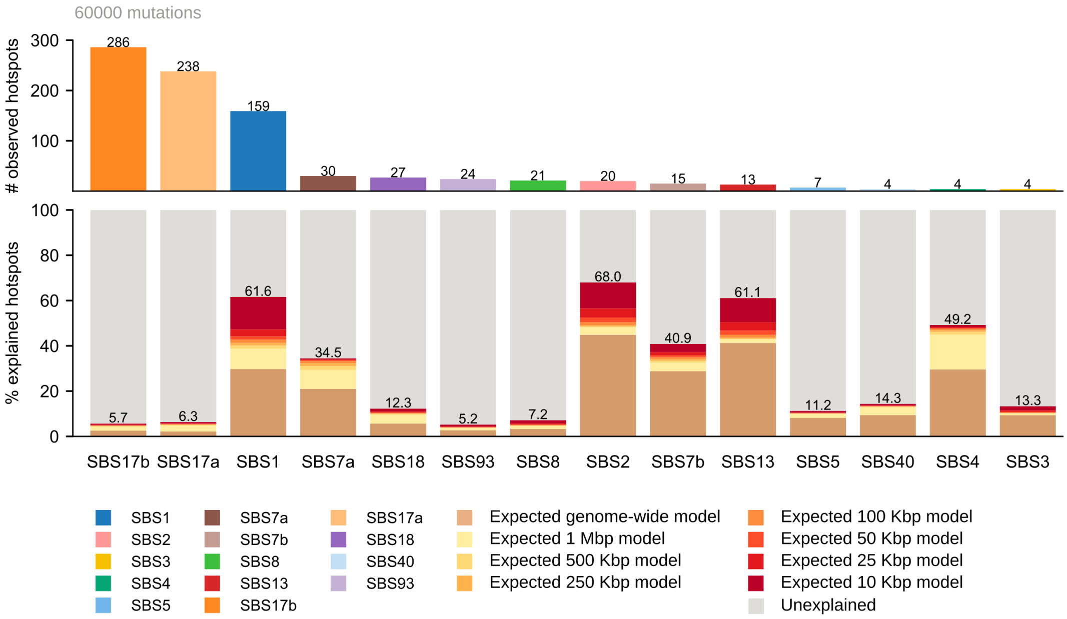

Appendix Figure S25. Expected hotspot propensity with larger subsampling. Median number of hotspots per signature observed by subsampling 60,000 total mutations (600 mutations/sample, 100 samples) (top). Percentage of explained hotspots by the different models (bottom). Note that the number of cancer types in this experiment differs from that shown in Fig 4F where a subsampling of 30,000 total mutations (300 mutations/sample, 100 samples) was used. Specifically, the following signature-cancer type pairs are not included in this experiment do to insufficient sampling size: SBS1-BLADDER_URI, SBS1-BRCA, SBS1-NSCLC, SBS5-BLADDER_URI, and SBS17a-COADREAD.

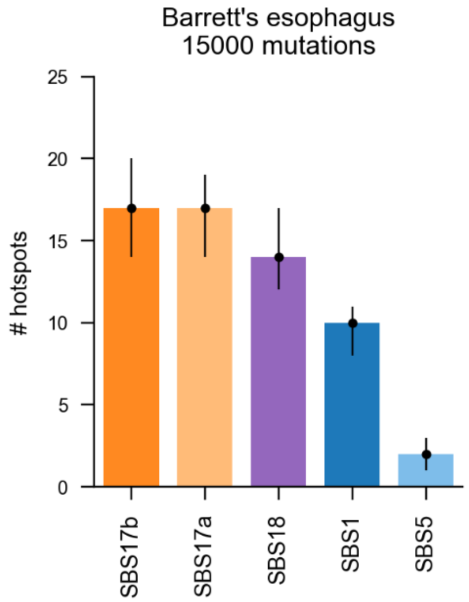

Appendix Figure S26. Hotspot propensity in Barrett’s esophagus. Hotspot propensity in Barrett’s esophagus samples was computed using a total of 15,000 mutations per signature (50 samples and 300 mutations/sample). Black dots show the median hotspot propensity across 1,000 subsamples; error bars represent the IQR.

Appendix Note 1
HotspotFinder

HotspotFinder is an algorithm implemented in Python 3.6 to identify and annotate hotspots of somatic mutations across the hg38 human genome. HotspotFinder identifies recurrently mutated genomic positions (in two or more samples) across the genome. Then, it annotates hotspots with a set of genomic data to contribute to their interpretation.

HotspotFinder v1.0 freely available at: `bitbucket.org/bbglab/hotspotfinder`

1. Input data

The minimum required HotspotFinder input consists of a file containing single nucleotide variants (SNVs), multiple nucleotide variants (MNVs) and/or small insertions and deletions (indels). The data to annotate hotspots is automatically downloaded within the package installation. Alternatively, users can provide their own paths to genomic annotations as described below.

Mutations

HotspotFinder's main input file is a TSV or CSV file containing SNVs, MNVs and short indels from a cancer cohort generated by whole genome (WGS), whole exome (WXS) or panel sequencing. In the current version, mutations are supported in the human genome build hg38. The mutations file requires the following information: chromosome, position, reference nucleotide(s), alternate nucleotide(s) and sample identifier. The header of the mutation file is 'CHROMOSOME', 'POSITION', 'REF', 'ALT', 'SAMPLE'.

By default, hotspots are identified using all samples in the mutations file. Alternatively, HotspotFinder can report hotspots independently in sub-groups or categories drawn from the input file. This feature requires adding an extra column with one of these headers: 'GROUP', 'GROUP_BY', 'COHORT', 'CANCER_TYPE', 'PLATFORM', and 'TYPE'. The new column name must be specified in the configuration file.

Genomic annotations

HotspotFinder incorporates genomic information to annotate hotspots and help in their interpretation. These annotations include: genome mappability, repeats and low complexity regions, population variants, immunoglobulin loci, and genomic elements (coding sequences from protein coding transcripts, splice sites, introns, 5' and 3' untranslated regions, promoters, non-coding RNAs, and pseudogenes). These files are automatically downloaded from bgdata package the first time that HotspotFinder is run. Users can also replace the automatic annotations with their own data by adding paths to the files within the configuration file:

- *Genome mappability.* Mappability was annotated from two different sources: regions of high mappability in the hg38 reference genome build and hg38 blacklisted regions of low mappability. In order to obtain high mappability regions, The GEnomic Multi-tool (GEM) (Marco-Sola *et al*, 2012) mappability software version 2013-04-06 was run on hg38 and those sequences with 100-mer pileup mappability equal or greater than 0.9 were kept. Blacklisted mappability regions were obtained from ENCODE (ENCODE Project Consortium, 2012) Unified GRCh38 Blacklist (downloaded from encodeproject.org/files/ENCFF356LFX on 16-06-2020).
- *Population variants*. Population variants were obtained from gnomAD (Karczewski *et al*, 2020) (downloaded from gnomad.broadinstitute.org on 25-06-2020) version 3.0. We kept variants with an allele frequency equal or greater than 1% (polymorphisms).
- *Repeats and low complexity regions.* Repeats across hg38 genome computed by RepeatMasker (RepeatMasker open-4.0.5, Repeat Library 20140131, hg38, Dec 2013) (developed by Smit, Hubley, Green at repeatmasker.org) were downloaded on 19-11-2020 at repeatmasker.org/species/hg.html
- *Immunoglobulin loci.* Immunoglobulin variable chain and T-cell receptor genes in hg38 build were obtained from Gencode (Frankish *et al*, 2019) v35 comprehensive gene annotation file at ftp.ebi.ac.uk/pub/databases/gencode/Gencode_human/release_35/gencode.v35.annotation.gtf.gz (downloaded on 30-08-2020).
- *Genomic elements*. The following genomic elements from hg38 build were defined from Gencode (Frankish *et al*, 2019) v35 comprehensive gene annotation file at ftp.ebi.ac.uk/pub/databases/gencode/Gencode_human/release_35/gencode.v35.annotation.gtf.gz (downloaded on 30-08-2020). Annotations were generated for all transcripts per gene, including the following genomic element categories.

- Coding sequences (CDS): protein-coding sequences within protein-coding transcripts.
- Splice sites: first and last 25 bp sequences within introns of protein-coding transcripts.
- 5'UTRs: 5' untranslated sequences from protein-coding transcripts.
- 3'UTRs: 3' untranslated sequences from protein-coding transcripts.
- Proximal promoters: sequences 200 bp 5' and 200 bp 3' of transcription start sites (inclusive) of protein coding transcripts. These sequences may overlap 5'UTRs or CDS sequences.
- Distal promoters: sequences 2,000 bp 5' of transcription start sites of protein coding transcripts.
- Introns: intronic sequences of protein-coding transcripts.
- lncRNA exons: exonic sequences of long non-coding RNA genes.
- lncRNA splice sites: first and last 25 bp sequences within introns of long non-coding RNA genes.
- lncRNA proximal promoters: sequences 200 bp 5' and 200 bp 3' of transcription start sites (inclusive) of long non-coding RNA genes.
- lncRNA distal promoters: sequences 2,000 bp 5' of transcription start sites of long non-coding RNA genes.
- lncRNA introns: intronic sequences of long non-coding RNAs.
- sncRNA: small non-coding RNAs of different types, including mt_rRNA, mt_tRNA, miRNA, misc_RNA, rRNA, scRNA, snRNA, and snoRNA. A complete description of each category is available at gencodegenes.org.
- Pseudogenes: all pseudogenes annotated at gencodegenes.org.

Considering that genomic elements may overlap each other, we set a hierarchical system of annotation priorities based on the potential functional relevance to protein-coding sequences. From highest to lowest priority, the overlap hierarchy was: CDS, splice sites, UTRs, promoters, introns, and lncRNAs (including their exons, introns and promoters) other ncRNAs and pseudogenes. No particular priority was assigned to the elements within the last three categories (their annotations are assigned equal priority and thus are allowed to overlap each other).

Configuration file

HotspotFinder allows a wide range of configurations to run. These can be specified through `hotspot.conf` file.

2. Output data

HotspotFinder generates two output files: `results.tsv` and `warningpositions.txt`

HotspotFinder hotspots

The main output of HotspotFinder is saved in the TSV file `results.tsv`. Each row in the results file corresponds to a hotspot. Hotspots are independently identified in each of the four mutation types: single nucleotide variants (SNVs), multi-nucleotide variants (MNVs), short insertions and short deletions.

By default, `results.tsv` file contains the following columns:

- CHROMOSOME: chromosome.
- POSITION: position.
- CHR_POS: chromosome and position.
- HOTSPOT_ID: unique hotspot identifier.
- MUT_TYPE: mutation type.
- COHORT: input cohort (file) name.
- N_MUTATIONS: number of mutations in the hotspot.
- N_MUTATED_SAMPLES: number of mutated samples in the hotspot.
- FRAC_MUTATED_SAMPLES: fraction of mutated samples in the cohort.
- REF: reference nucleotide.
- ALT: alternate nucleotide(s).
- ALT_COUNTS: number of alternate nucleotide(s).
- FRAC_ALT: proportion of alternates among total alternates.
- CONTEXT_3: trinucleotide context centred at the hotspot.
- CONTEXT_5: pentanucleotide context centred at the hotspot.
- N_COHORT_SAMPLES: number of samples in the cohort.
- N_COHORT_MUTATIONS_TOTAL: number of total mutations in the cohort.
- N_COHORT_MUTATIONS_SNV: number of single base substitutions in the cohort.

- N_COHORT_MUTATIONS_MNV: number of multi-nucleotide substitutions in the cohort.
- N_COHORT_MUTATIONS_INS: number of insertions in the cohort.
- N_COHORT_MUTATIONS_DEL: number of deletions in the cohort.
- MUTATED_SAMPLES: identifier of mutated samples.
- MUTATED_SAMPLES_ALTS: identifier and corresponding alternate of mutated samples.
- OVERLAP_WARNING_POSITION: True if the hotspot overlaps a warning position.

If HotspotFinder is run with annotations, additional columns are included in `results.tsv` file:

- GENOMIC_ELEMENT: overlapping genomic element(s)
- SYMBOL: symbols off overlapping genomic element(s)
- GENE_ID: Ensembl gene identifiers of overlapping genomic elements
- TRANSCRIPT_ID: Ensembl transcript identifiers of overlapping genomic elements
- GENOMIC_REGION: class of overlapping genomic element(s)
- GENOMIC_REGION_PRIORITY: genomic element class with highest priority
- CODING_NONCODING: overlapping coding or non-coding genomic elements
- MAPPABILITY: high or low according to GEM mappability regions overlap
- MAPPABILITY_BLACKLIST: pass if no overlap with blacklisted mappability regions
- VARIATION_AF: overlapping population variants
- HOTSPOTFINDER_FILTERS: flag if hotspot is mappable and does not overlap blacklisted mappability regions or population variants.
- REPEATS: overlapping repeats.
- REPEATS_OVERLAP: flag if hotspot overlaps repeats.
- IG_TR: overlapping immunoglobulin regions.
- IG_TR_OVERLAP: flag if hotspot overlaps immunoglobulin regions.

HotspotFinder warning positions

During the process of mutation parsing some unexpected data can be observed and are reported in a separate TSV file, `warningpositions.txt`, with the following header: 'CHROMOSOME', 'POSITION', 'REF', 'ALT', 'SAMPLE', 'WARNING', 'SKIP' (check “Mutation parsing” for further information).

3. Implementation

Mutation parsing

Parsing of somatic mutations is performed in two steps as explained below:

In the first step, the package `bgparsers` reads the mutation file and categorises mutations into 3 mutation types: SNVs, MNVs and indels. Indels are further classified into simple insertions (e.g., G>GA or G>GAAA) or simple deletions (e.g, GT>G, GTG>G). Complex indels that are a mixture of both previous categories (e.g., GTG>GA, GTG>GAAA) are skipped. At this level, some filters are applied: 1) mutations falling outside of autosomal or sexual chromosomes are skipped; 2) mutations having equal reference and alternate nucleotides are removed; 3) mutations with annotated reference nucleotide(s) that do not correspond to the reference genome build (in this case, only for SNVs, MNVs and deletions) are discarded; 4) mutations that contain an unknown nucleotide (a nucleotide not corresponding to A, C, G, T) are removed; 5) mutations with an unknown nucleotide in their pentamer sequence context, according to their start position, are skipped from the analysis.

In the second step, the algorithm checks how many alternates a given sample (patient) has for each mutated position of the genome. It is expected that homozygous sample for a locus will have a single alternate (e.g., chr1:23798511_G>A), whereas heterozygous samples can have 2 (e.g., chr1:23798511_G>A and chr1:23798511_G>T). However, other scenarios may arise such as homozygous samples annotated with two equal alternates separately in the input file, heterozygous samples bearing 3 different alternates in the case of SNVs or more than 3 in the case of other mutations. Given that the algorithm is agnostic of how the mutation calling has been performed and additional information that might be of interest to interpret these situations such as tumour ploidy or copy number alterations, it parses mutations using the following criteria:

- If there is only one alternate count for a given patient, genome position and mutation type, this mutation is kept for the analysis.
- If there is more than one alternate count for a given patient, genome position and mutation type, three situations can occur and are categorised in a warning level as follows: 1) a unique alternate is found more than one time (e.g., chr1:23798511_G>A and chr1:23798511_G>A); 2) there are two different alternates (e.g.,

chr1:23798511_G>A and chr1:23798511_G>C); 3) there are three or more different alternates (e.g., chr1:23798511_G>A, chr1:23798511_G>C and chr1:23798511_G>T). Given that mutations in scenario 1 and 2 are likely to reflect *bona fide* biological information (homozygous or heterozygous mutants for a locus, respectively) all mutations are kept for analysis. This implies that the number of mutations and the number of mutated samples in a given position in the genome will not match. Mutations in scenario 3 are discarded because their biological interpretation is unclear considering the data available within HotspotFinder. Any mutation falling in these three categories is reported in the output file `warningpositions.txt`. This information is also highlighted in the 'OVERLAP_WARNING_POSITION' column in the hotspots output file `results.tsv`.

Hotspots identification

Hotspots are defined as single genomic positions bearing 2 or more somatic mutations from 2 or more different samples. Hotspots are independently identified for 4 types of mutations: single nucleotide variants (SNVs), multi-nucleotide variants (MNVs), short insertions and short deletions.

By default, mutations are merged together according to their reference start position, irrespective of the length of the reference or alternate nucleotides in the case of MNVs, insertions and deletions. For instance, the variants chr1:123456_A>T mutation, chr1:123456_A>T, and chr1:123456_A>C mutation will be identified as a hotspot of SNVs in the position chr1:123456 with alternates T,T,A. In the case of MNVs, variants are merged into hotspots if they start in the same genomic position. For example: if the variants chr17:7571726_GTG>CCC, chr17:7571726_GT>CA, chr17:7571726_GTG>CCA appear in three independent samples, they will be merged into a hotspot. In the case of insertions, chr1:123456_A>AT, chr1:123456_A>ATT and chr1:123456_A>AGG variants in three independent patients will result in an insertion hotspot at chr1:123456 with alternates T,TT,GG. Similarly, in the case of deletions, chr1:123456_AG>A, chr1:123456_AGT>A and chr1:123456_AGTA>A mutation will result in a hotspot at chr1:123456 with reference sequences G, GT and GTA.

In the current version, it is also possible to restrict the identification of hotspots to those mutations sharing the same nucleotide change in the case of SNVs and MNVs, equal inserted sequence in the case of insertions or equal deleted sequence in the case of deletions. This behaviour can be specified in the configuration file. All results shown within the current manuscript are based on alternate-specific hotspots. This option was chosen to increase the signal-to-noise ratio to further analyse the mutational processes causing hotspots.

Hotspots annotation

Once hotspots are identified, by default HotspotFinder intersects their coordinates with genomic annotations. One can disable this option in the configuration file and the running time will be shortened. Hotspots that do not overlap high mappability regions or do overlap mappability blacklisted regions are considered artefact hotspots and are flagged accordingly. Similarly, hotspots that overlap a polymorphic site, irrespective of the mutation type or alternate, are considered artefacts of germline filtering. Importantly, these hotspots are kept in the output file `results.tsv`. By default, hotspots that do not overlap genomic elements are removed from the output file. This behaviour can be changed in the configuration file.

Appendix Note 2
Extraction of mutational signatures

Mutational signatures extraction using total SNVs

We extracted *de novo* trinucleotide-context single base substitution signatures (96-mutation types using pyrimidines as reference) using SigProfiler framework (Alexandrov *et al*, 2013b; Islam *et al*, 2022; Bergstrom *et al*, 2019) over the 31 cancer types, excluding Pancancer, with at least 30 samples and 100,000 total SNVs (hotspot and non-hotspot mutations): ALL, BILIARY_TRACT, BLADDER_URI, BONE_SOFT_TISSUE, BOWEL, BRAIN, BRCA, CERVIX, COADREAD, ES, ESOPHA_STOMACH, GBM, HEAD_NECK, KIDNEY, LIVER, LNM, LUNG, MBL, NHL, NSCLC, OV, PANCREAS, PLMESO, PROSTATE, SBNET, SCLC, SKCM, SKIN, ST, THYROID, UCEC.

For each cancer type, GRCh38 96-mutational catalogues of total SNVs were calculated using SigProfilerMatrixGenerator (Bergstrom *et al*, 2019) version 1.1.26. Mutational signatures were extracted with SigProfilerExtractor (Islam *et al*, 2022) version 1.1.0 testing the optimal solution for minimum of 1 and a maximum of 20 signatures (minimum_signatures, maximum_signatures parameters) with 1,024 NMF replicates (nmf_replicates parameter) and GRCh38 reference genome. All other NMF parameters were set as default. Solutions with average signature stability above 0.8, minimum signature stability above 0.2 and mean cosine distances (error) below 0.15 were pre-selected for manual review. We then assessed the cosine similarity between them and COSMIC v3.2 reference signatures by computing the complementary of the distance among each pair of vectors of mutational probabilities using scipy.spatial.distance:

$$cosine\ similarity = 1 - cosine\ distance$$

De novo signatures were assigned to the best matching (largest cosine similarity) COSMIC reference signature. After reviewing the assigned signature sets among all solutions, the best solution was selected based on the biological plausibility of the extracted signatures. When different solutions showed equally plausible assignments by cosine similarity, the solution with smallest mean cosine distances and largest cosine similarity was selected (Appendix Note 2 Fig 1).

In order to compare signatures activity across cancer types, the best solution was finally decomposed into COSMIC v3.2 reference signatures using the default decomposition function within SigProfilerExtractor (refit_denovo_signatures=True). Signatures that could not be decomposed, and therefore were not equivalent among cancer types, were discarded from further analysis. Cancer types whose signature extractions had no solution matching the above-mentioned quality criteria were discarded for further analysis, including BONE_SOFT_TISSUE, LNM, NBL, and NHL. Likewise, the following known artefact signatures (Alexandrov et al, 2020) were excluded from the analysis: SBS27, SBS43, SBS45, SBS46, SBS47, SBS48, SBS49, SBS50, SBS51, SBS52, SBS53, SBS54, SBS55, SBS56, SBS57, SBS58, SBS59, SBS60.

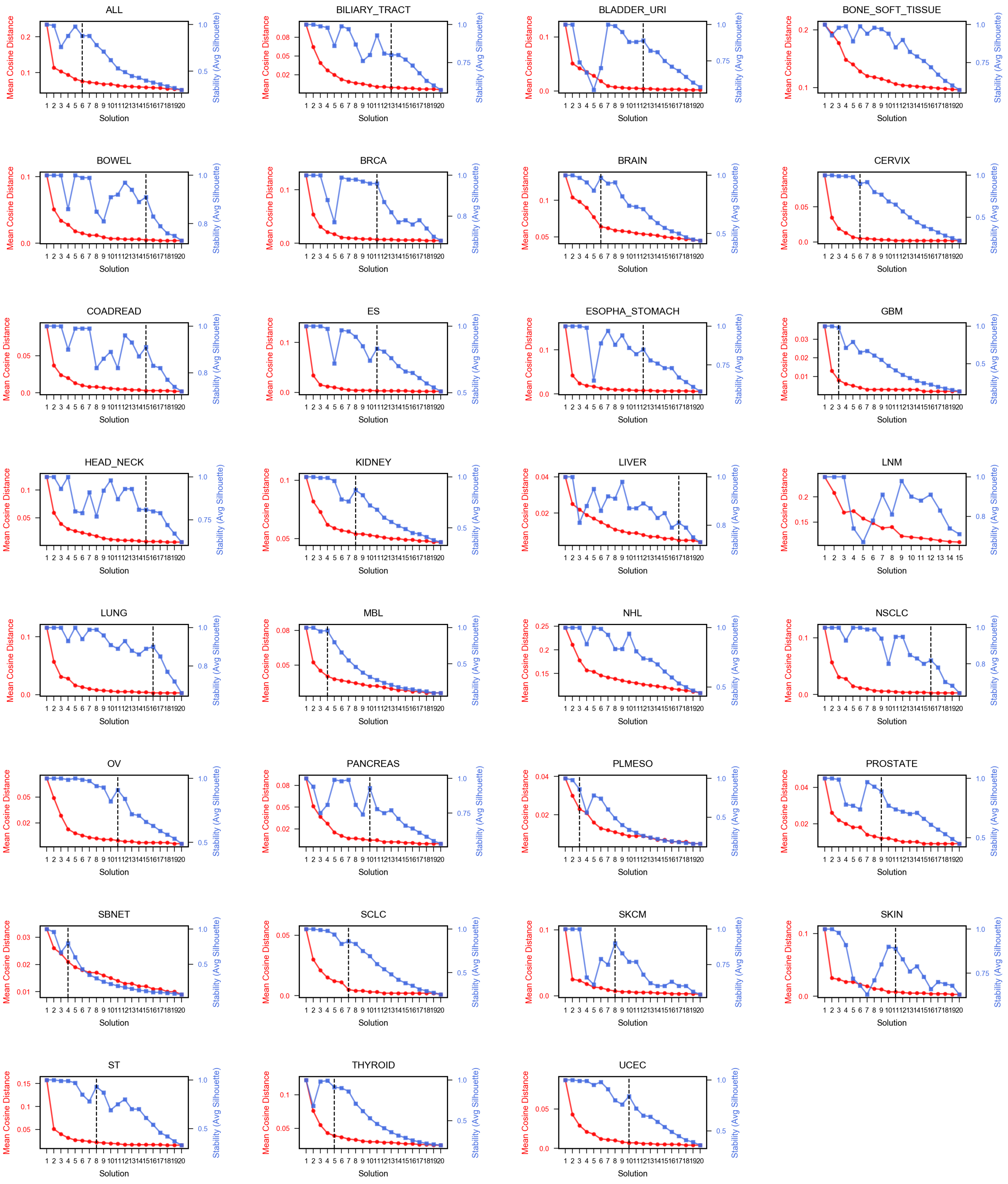

Appendix Note 2 Figure 1. Selection plots for extracted signatures using total SNVs. Vertical dashed lines show the best extracted solution that fulfilled the quality criteria explained above.

Extractions of SBS17a and SBS17b

SBS17a and SBS17b are two signatures with high hotspot propensity in our analysis, both of which are of unknown aetiology (Alexandrov *et al*, 2020). SBS17a is characterised by frequent CpTpN>C transitions, while SBS17b preferentially shows NpTpT>G transversions. CpTpT trinucleotides are the most frequently mutated in both of them (Alexandrov *et al*, 2020). First identified as a joint signature, SBS17 (Alexandrov *et al*, 2013a), these signatures have been found in primary tumours, notably in esophageal cancers (Secrier *et al*, 2016), and, in the case of SBS17b, also in metastases of patients exposed to 5-fluorouracil (5-FU) or capecitabine during the treatment or their primary tumours (Pich *et al*, 2019; Christensen *et al*, 2019).

In our study, SBS17a and SBS17b were found across esophageal-stomach and colorectal cancers (Appendix Fig S12). Three *de novo* signatures contributed to SBS17a and SBS17b in each cancer type (Appendix Note 2 Fig 2). Among both sets, the signatures contributing to more mutations (SBS96-A in esophageal-stomach and SBS96-F in colorectal cancers) showed both the T>G and T>C components of SBS17b and SBS17a, with predominance of T>G transversions that explain their higher cosine similarity to COSMIC v3.2 SBS17b). Additionally, two signatures (SBS96-B in esophageal-stomach and SBS96-I in colorectal cancers) were clearly identified as SBS17b (cosine similarity of 1 and 0.97 to SBS17b, respectively). Among the 194 esophageal-stomach tumours with SBS96-B activity (defined by a signature contribution of at least 5% of mutations of the total mutation burden), 112 were metastatic and 37 (33%) of these had been exposed to either 5-FU or capecitabine. In colorectal cancers, 146/150 tumours bearing SBS96-B were metastases, the majority of which (n=123, 84%) showed 5-FU or capecitabine exposure. This is consistent with previous studies on SBS17b (Pich *et al*, 2019; Christensen *et al*, 2019). Altogether, these data explain the larger mutation burden of SBS17b compared to SBS17a in our dataset.

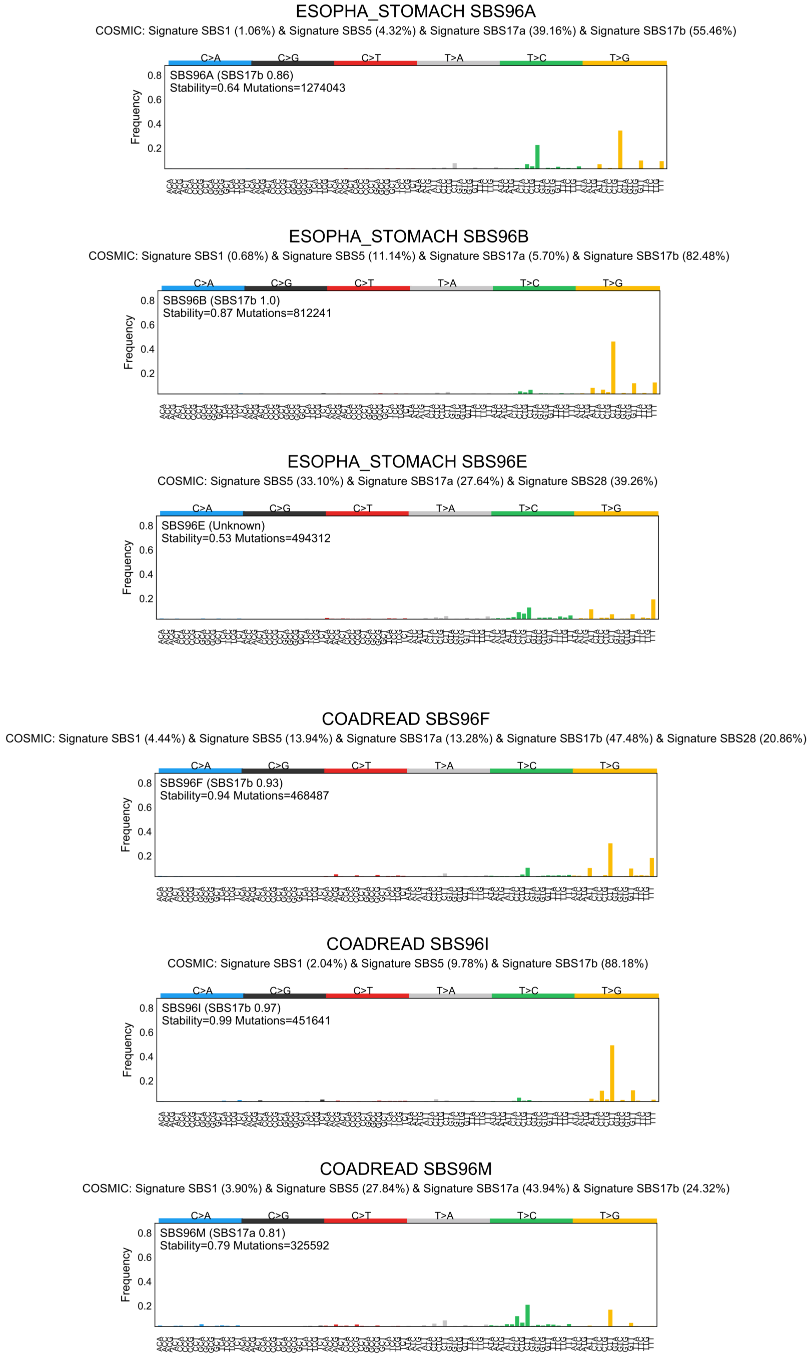

Appendix Note 2 Figure 2. Extracted signatures contributing to SBS17a and SBS17b. *De novo* extracted signatures partially decomposed into COSMIC v3.2 SBS17a and SBS17b signatures. The signature decomposition attribution to these and other signatures is shown.

Mutational signatures extraction using SNVs inside hotspots

A second extraction of *de novo* trinucleotide-context single base substitution signatures (96-mutation types using pyrimidines as reference) was carried out using exclusively the set of SNVs inside hotspots for the top 4 cancer types with largest number of hotspots: COADREAD; ESOPHA_STOMACH, NSCLC, SKCM. GRCh38 96-mutational catalogues of SNVs overlapping hotspots were calculated using SigProfilerMatrixGenerator (Bergstrom *et al*, 2019) version 1.1.26. Mutational signatures were extracted with SigProfilerExtractor (Islam *et al*, 2022) version 1.1.0 running for a minimum of 1 and a maximum of 15 signatures (minimum_signatures, maximum_signatures parameters) with 1,024 NMF replicates (nmf_replicates parameters). All other NMF parameters were set as default. The best solution was selected following the same approach as in the total SNVs extraction (Appendix Note 2 Fig 3). The resulting *de novo* signatures inside hotspots listing the COSMIC v3.2 signature with largest cosine similarity above 0.8 are shown below (Appendix Note 2 Fig 4)

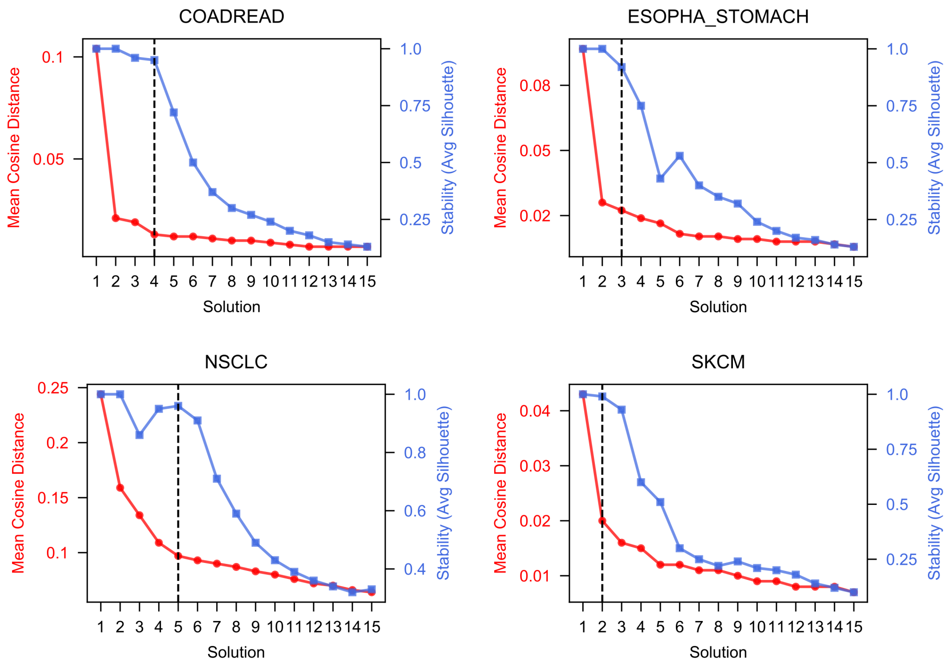

Appendix Note 2 Figure 3. Selection plots for extracted signatures using SNVs inside hotspots. Vertical dashed lines show the best extracted solution that fulfilled the quality criteria explained above.

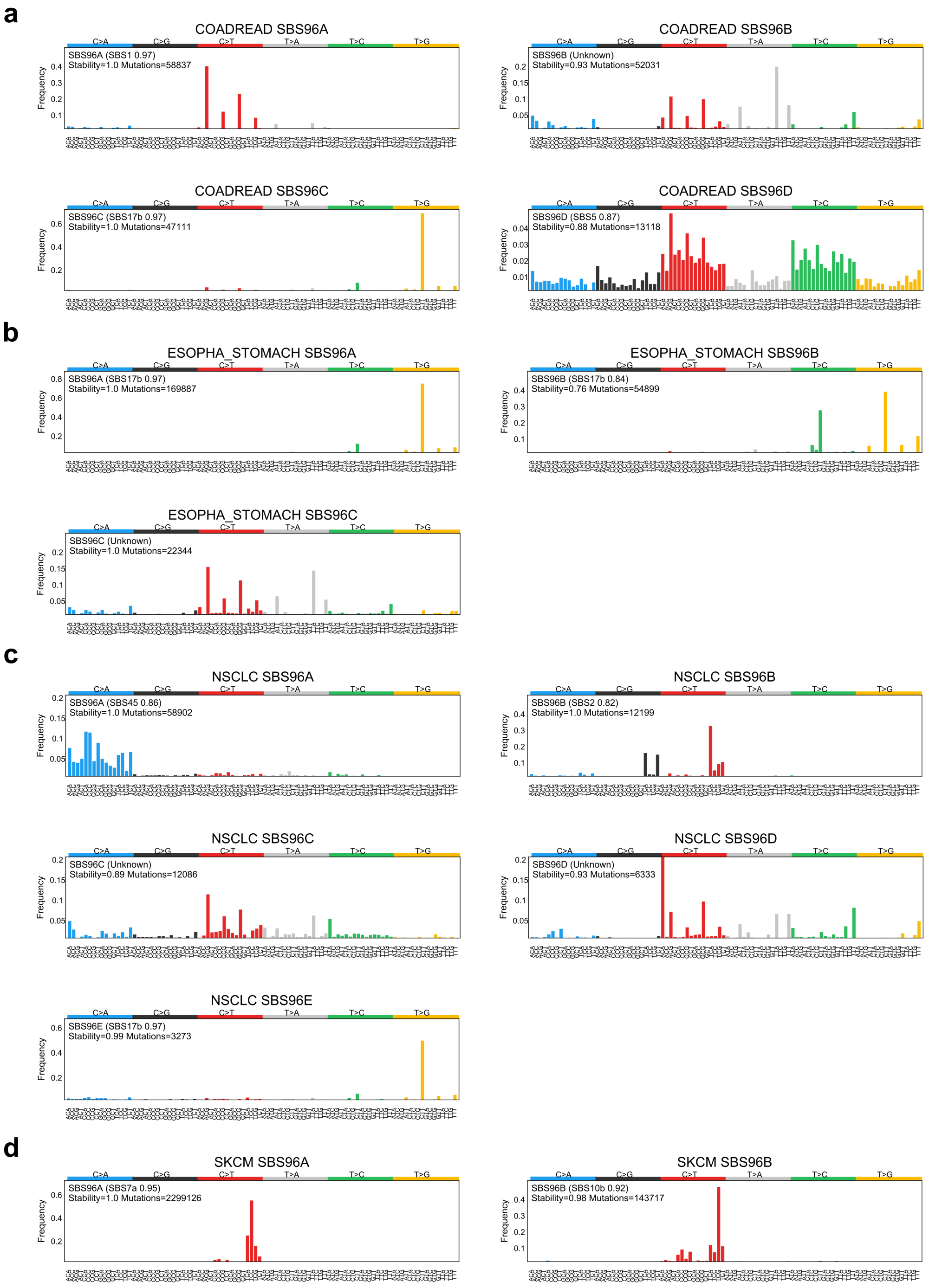

Appendix Note 2 Figure 4. Extracted mutational signatures from SNVs inside hotspots. Signature profiles showing the *de novo* extracted signatures for **a**) colorectal cancers, **b**) esophagus-stomach cancers, **c**) non-small cell lung cancers and **d**) skin melanomas. The COSMIC signature with largest cosine similarity above 0.8 to the profile is stated, otherwise it is labelled as unknown.

Appendix Note 3
Assignment of mutational signatures to mutations and hotspots

Cancer datasets

As part of our analysis in cancer samples, we assigned or attributed mutations and hotspots to the mutational signature that most likely generated them. This attribution was carried out as follows.

As detailed in Appendix Note 2, we first extracted *de novo* mutational signatures from cancer types using SigProfilerExtractor (Islam *et al*, 2022). In order to obtain an homogeneous set of reference signatures and their exposures across cancer types, we then used SigProfilerExtractor decomposition of *de novo* signatures into the COSMIC v3.2 signatures reference set. Specifically, SigProfilerExtractor uses a nonnegative least squares (NNLS) algorithm to identify the coefficients (contributions) of COSMIC signatures that best recapitulate each of the *de novo* signatures in a cancer type (for details see Islam *et al*, 2022). Once the contribution of a set of COSMIC signatures has been obtained for each *de novo* signature, an additional NNLS algorithm is applied to identify the activity of COSMIC signatures to each sample in the dataset. Then, the probability of a particular mutation within a sample (attributed to a trinucleotide-context substitution, e.g., A[C>A]A) to arise from a COSMIC decomposed signature is calculated based on the mutation trinucleotide-context, the sample-specific signature exposures and the signature profile (mutational probabilities per trinucleotide-context).

Specifically, the likelihood that signature S contributes to a mutation observed in triplet context t and sample k is computed as follows (Morganella *et al*, 2016):

$$P(S;t,k)=\frac{w_{km}S(t)}{\sum_{i=1}^{M} w_{ki}S_i(t)}$$

where the vector of total exposures of the sample is given by the following weighted sum:

$$e_k=\sum_{i=1}^{M} w_{ik}S_i$$

S(t) denotes the frequency of context t in the profile of signature S and i indexes all the mutational signatures contributing mutations to the cohort.

This results in a vector of mutational probabilities per sample and trinucleotide context across the active signatures in the cancer type. From this matrix of vectors, we then assign individual mutations within a sample to a mutational signature using maximum likelihood, if the probability of maximum likelihood signature is above a probability of 0.5 (Appendix Note 3 Fig 1). The mutational profiles resulting from mutations assigned by maximum likelihood to a signature showed a high similarity to the corresponding COSMIC signature profile (median cosine similarity = 0.98, range=(0.63, 1); Appendix Note 3 Fig 2).

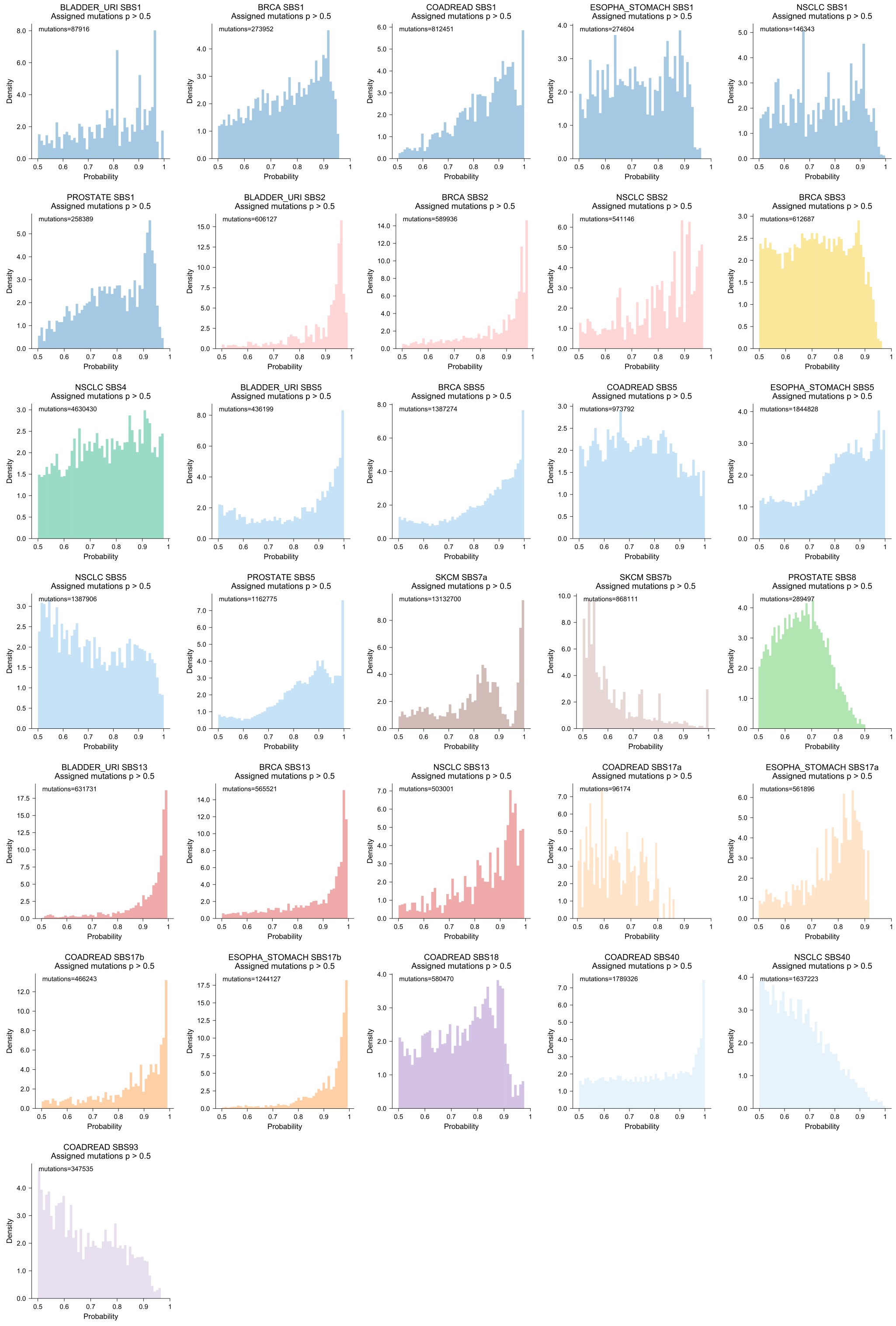

Appendix Note 3 Figure 1. Distribution of mutational probabilities across mutations attributed to a signature. Histograms showing the distribution of mutational probabilities for mutations attributed to COSMIC mutational signatures by maximum likelihood (p > 0.5) across cancer types.

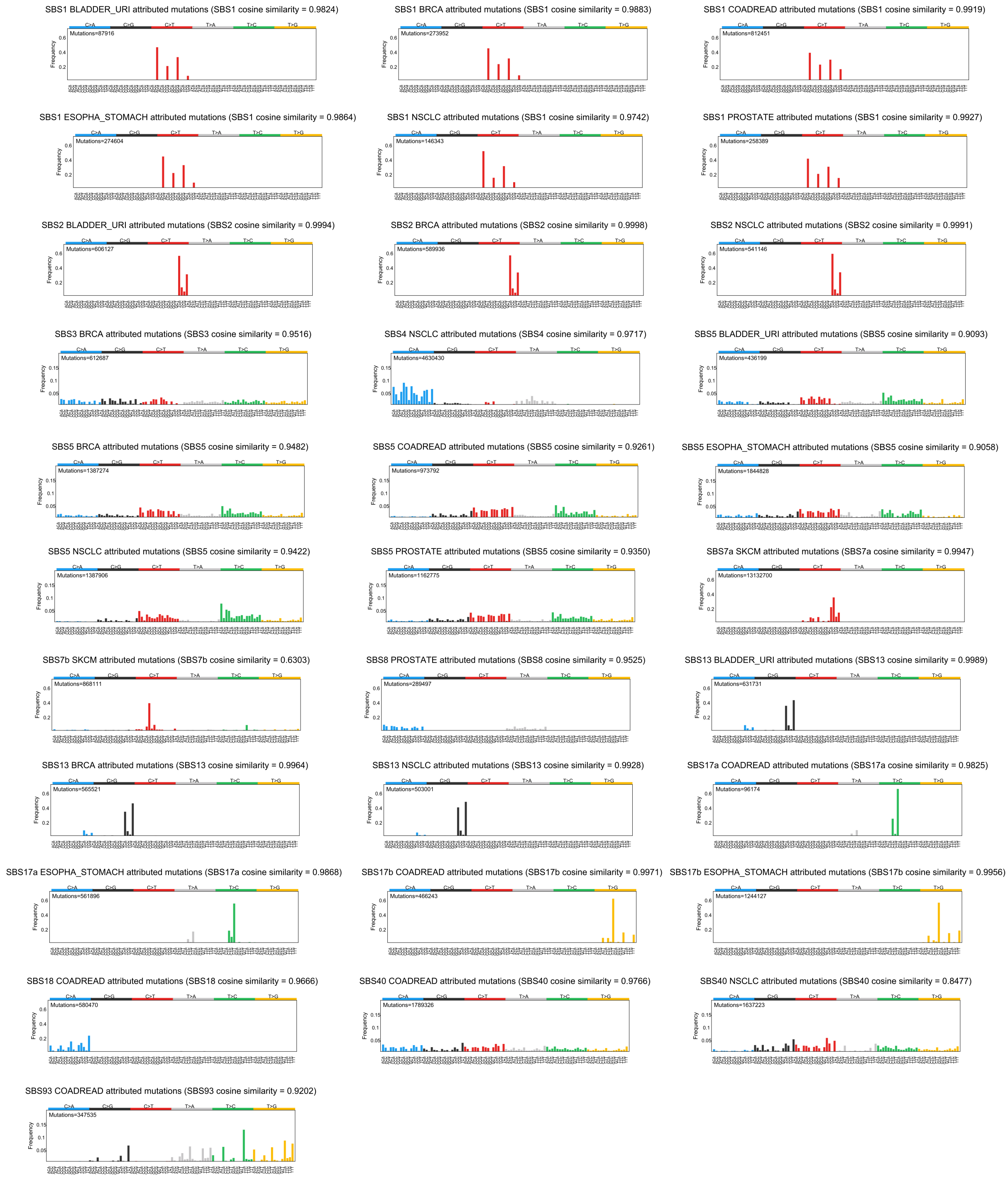

Appendix Note 3 Figure 2. Mutational profiles of mutations attributed to signatures across cancers. Mutational profiles for mutations attributed to COSMIC mutational signatures by maximum likelihood ($p > 0.5$) across cancer types. The cosine similarity to the corresponding COSMIC signature is shown on top.

Normal tissue datasets

Similarly as in the cancer datasets, we attributed mutations in colonic crypts (Cagan *et al*, 2022), Barrett's esophagus (Paulson *et al*, 2022) and germline datasets (Rahbari *et al*, 2016; An *et al*, 2018; C Yuen *et al*, 2017; Sasani *et al*, 2019; Halldorsson *et al*, 2019; Goldmann *et al*, 2016) to mutational signatures active in the respective tissue by maximum likelihood with a probability above 0.5. The mutational profiles of the resulting attributed mutations per signature showed a high cosine similarity (> 0.9) to their reference COSMIC signature (Appendix Note 3 Fig 3).

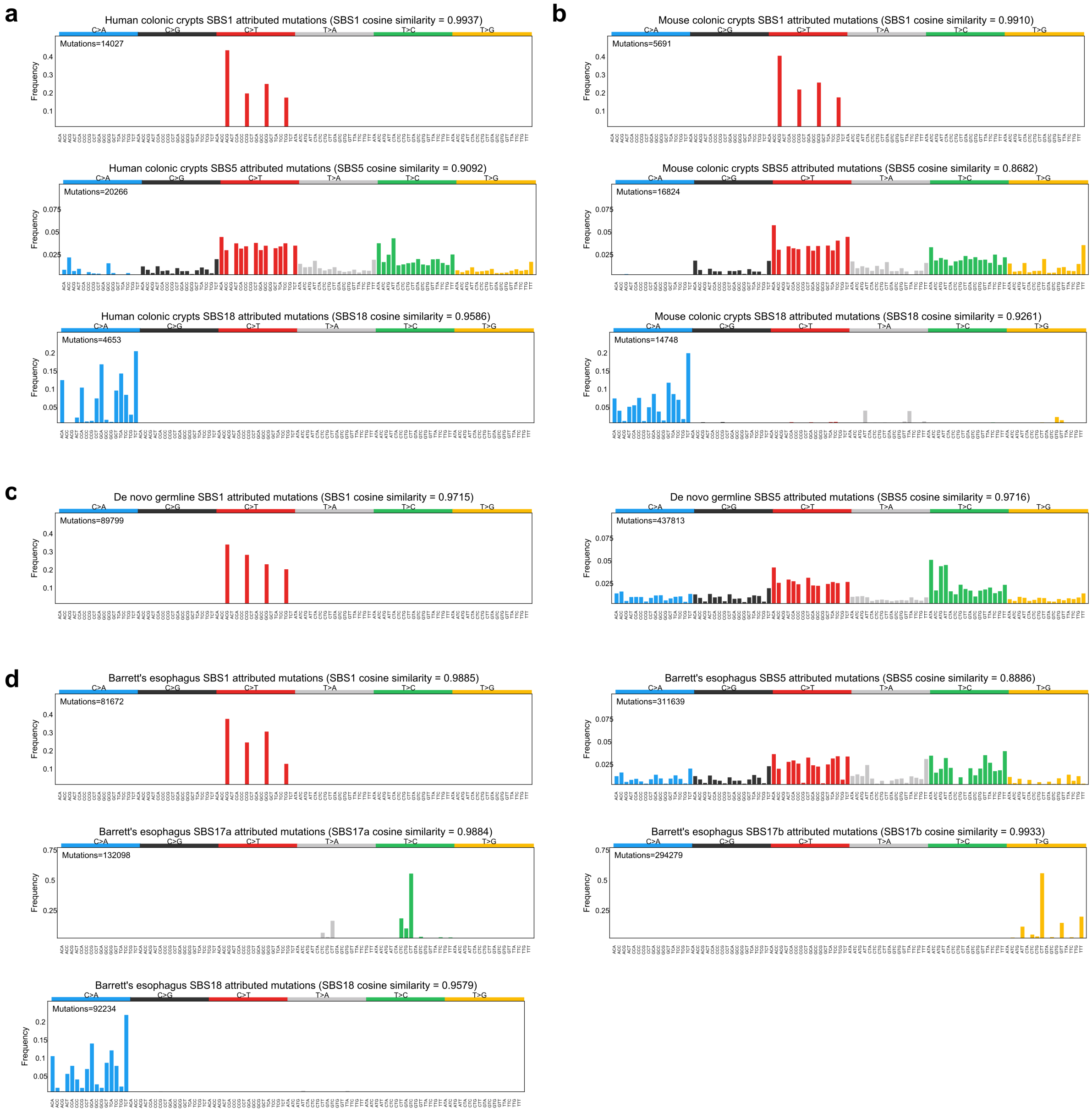

Appendix Note 3 Figure 3. Mutational profiles of mutations attributed to signatures in normal tissues. Mutational profiles for mutations attributed to COSMIC mutational signatures by maximum likelihood ($p > 0.5$) in **a**) human colonic crypts, **b**) mouse colonic crypts, **c**) de novo germline mutations and **d**) Barrett's esophagus. The cosine similarity to the corresponding COSMIC signature in the matched reference genome is shown on top.

Appendix Note 4
Comment on hotspot propensity

We used two metrics to calculate the propensity of mutational processes to form hotspots. First, we calculated the number of newly observed hotspots through subsampling of mutations contributed by a signature under equal mutation and sample sizes across a cohort (Fig 2C-F). Briefly, for each cancer type, we assigned mutations to the signature with maximum probability of having generated them. To increase the accuracy of this allocation, we only attributed a mutation to a signature if its probability was above 0.5. Then, we randomly selected (subsampled) 1,000 sets of N mutations (e.g., 30,000 mutations) from each signature across 100 patients (all of which contributed with equal mutation burden) and computed the number of hotspots observed in each subsample. Under equal mutation burden, we expect to observe more hotspots contributed by signatures with higher propensity to form them. Given that we leverage separate sets of mutations per signature, this estimate of a signature's hotspot propensity is independent of all other signatures in the cancer type. This is particularly important when evaluating mutational processes active in the same samples with overlapping trinucleotide preference, which may interact in the formation of hotspots. For example, SBS17a and SBS17b, which are active in esophageal and colorectal cancers, partially share their target trinucleotides (specially the CpTpT trinucleotide (Alexandrov *et al*, 2020)), but show different mutation burden in our dataset. By separating the sets of mutations attributed to each of them (Appendix Note 4 Fig 1), their hotspot propensities can be assessed independently from each other. This metric, however, assumes that mutations contributed by a signature are equally distributed across the tumours in a cohort. Since this is not necessarily true, we developed a metric that is orthogonal to the one described above.

In this second metric, we leveraged the total number of observed hotspots and mutations (classified as being outside or inside hotspots) contributed by all signatures and computed the fold change of the activity of each of them inside and outside hotspot mutations (Fig EV1). We reasoned that signatures with higher propensity to form hotspots would be more frequently found in the set of mutations inside hotspots than in the set of mutations outside hotspots (Appendix Fig S17). Therefore, if we computed the ratio (fold change) of a signature activity inside versus outside hotspots, those with propensity to form hotspots would show a fold change larger than 1. Conversely, signatures with no particular propensity for hotspots

formation would show similar frequencies in both sets of mutations and consequently a fold change of 1. The fold change for the top three signatures with highest hotspot propensity based on subsampling, SBS17b, SBS17a and SBS1, was larger than 1 across a wide variety of cancers (Fig EV1), thus validating our first observations. However, this measure showed some caveats.

Although applicable to most signature-cancer type pairs in our dataset without the need of subsampling samples and mutations, the fold change is computed using hotspots that have been contributed by all signatures present in the cancer type. That is, the hotspots according to which mutations are classified as inside or outside are assumed to be generated by a combination of mutational signatures in the cancer type, given the trinucleotide context and the signatures activities in the sample. Each mutation is treated as a vector of probabilities of the mutation arising from the catalogue of signatures in the cancer type. Then, in order to compute the frequency inside hotspots of a given signature in a cancer type, the vector of mutational probabilities across all mutations inside hotspots is normalised to 1. Likewise, the same step is carried out for outside mutations. This allows the comparison of signature frequencies inside and outside hotspots (otherwise the ratio of the sum of probabilities would be smaller than 1, assuming the number of mutations outside hotspots outnumbers that of mutations inside). The fact that the fold change is computed using hotspots contributed by different signatures poses a limitation to accurately identifying the hotspot propensity of different signatures with similar propensities but different activities in a cancer type.

As introduced before, a clear example of this are SBS17a and SBS17b in esophageal-stomach and colorectal cancers. SBS17b showed bigger hotspot fold change than SBS17a (median 3.1 and 1.2 fold change across cancer types, respectively). However, when we calculated hotspot propensity through subsampling of mutations independently assigned to any of both signatures by maximum likelihood (Appendix Note 4 Fig 1), SBS17a and SBS17b showed very similar high propensity to form hotspots, generating, respectively, 78 and 72 hotspots across 30,000 mutations (Fig 2F). The fact that SBS17b contributed more mutations than SBS17a in both cancer types and that both signatures target the same trinucleotides, particularly CpTpT sites, increases the probability of identifying SBS17b inside hotspots in detriment of SBS17a when the fold change enrichments are computed. For all these reasons, we chose the subsampling strategy as the main method to estimate hotspot propensity across signatures.

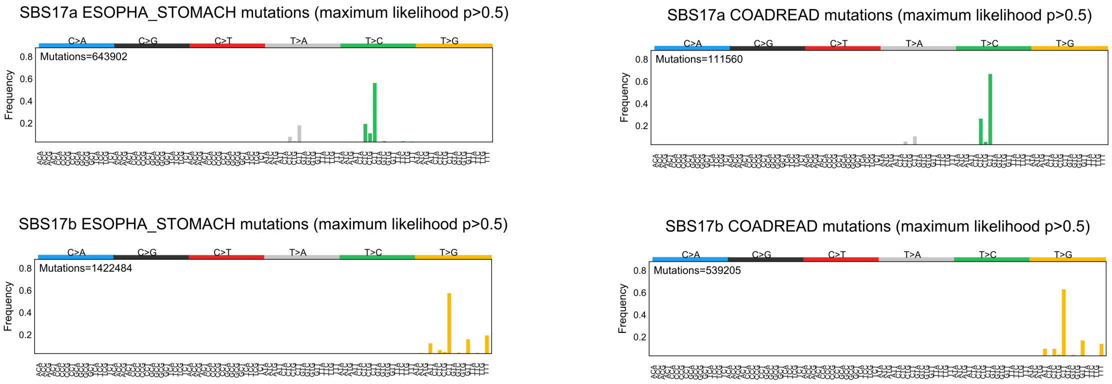

Appendix Note 4 Figure 1. Mutations assigned to SBS17a and SBS17b. Frequencies of trinucleotide-based substitutions (either inside or outside hotspots) attributed to SBS17a and SBS17b by maximum likelihood above 0.5.

Appendix Note 5
Theoretical models of hotspot propensity

Overview

Given a genomic chunk or region of DNA (e.g. the whole mappable reference genome or a specific covering of the genome in chunks of size 1 Mbp, 500, 250, 100, 50, 25 or 10 Kbp) we provide a theoretical calculation of the expected number of hotspots produced in it by a synthetic cohort of samples that have identical mutation rates at that region. We name this expected number of hotspots "hotspot propensity" when referring to the expected count at genome-wide level, which can be obtained by adding up the expected number of hotspots across genomic chunks of the given size.

We assume that mutations are generated in accordance with the same mutational process, which in this analysis will be characterised by the relative frequency of each nucleotide substitution taking into account the flanking nucleotides (trinucleotide context). Furthermore, in our computation we will make the simplifying assumption that mutations are generated independently across samples and genomic positions. Using this fact, note that if we partition a region into disjoint subregions, the expected number of hotspots should be equal to the sum of expectations across subregions. In particular, we can compute the expected number of hotspots at a single genomic position, then sum the expected number of hotspots across all genomic positions of the region of interest. Note that in our framework all positions with the same reference trinucleotide will bear the same expected number of hotspots if we assume that the mutation rate is homogeneous across positions.

Formally, given a genomic region, an homogeneous mutation rate and a mutational profile operative in the region, we can compute how many hotspots are expected at each position, which will depend on the reference trinucleotide of the position, then sum the expectations across positions to get a total expectation for the whole region.

In order to compute the expected number of hotspots at a single position, we first need to transform the overall mutation rate of the region into mutation probabilities at single positions. In the following sections we will explain in detail how to derive this mutation probabilities at single positions (section 1), how with this information we can derive the expected number of hotspots at a given position when stacking a cohort with several samples (section 2) and how we use this information to derive expected number of hotspots genome-wide (section 3) in mappable genomic segments (section 4) and how we include DNA methylation covariates (section 5).

Finally, in section 6 we showcase a stochastic generative model of hotspots counts that is compatible with our calculation of the expected hotspot rate.

1. Mutation probability at a single position

Consider a genomic region where a mutational process is operative with overall mutation rate μ, meaning that the expected number of mutations in the region is μ. Let $\underline{m}=(m_{t>a})$ be the vector of normalised mutation rates – indexed by pairs of reference triplet t and alternate allele a – that defines the mutational process. Here the term "normalised" implies that the vector already reflects the relative mutation rates across trinucleotide contexts after correcting for the trinucleotide content of the region where the mutational frequencies were sampled from. In other words, it reflects the relative likelihood to observe some trinucleotide contexts compared to others. For a position with reference triplet t we compute the probabilities to undergo either of the three possible alternate alleles (for simplicity denoted A, B and C) as follows:

$$p_{t>A}=\frac{1}{M}\mu m_{t>A},\quad p_{t>B}=\frac{1}{M}\mu m_{t>B},\quad p_{t>C}=\frac{1}{M}\mu m_{t>C},\quad p=p_{t>A}+p_{t>B}+p_{t>C}$$

$$\text{where}\quad M=\sum_{s=1}^{32} n_s\left(m_{s>A}+m_{s>B}+m_{s>C}\right)$$

and n_t is the number of positions in the region with reference triplet t. Note that for each triplet we can take A, B, C as e.g. the three possible alternate alleles with respect to the reference in lexicographic order (Appendix Note 5 Fig 1A).

2. Expected hotspot rate at a single position

Let's consider a stacked cohort of samples, each with identical mutation rates, and let's compute the expected number of hotspots at a specific genomic position. Let N denote the number of samples and p the probability that there is a mutation at a given sample. We will denote the possible alternate types at the position of interest as either A, B and C, and the respective per sample probabilities as p_A, p_B and p_C. Clearly, $p=p_A+p_B+p_C$.

A hotspot is called whenever the same alternate is found at the same position in at least two samples. We can think of the single position across samples as an N-word of base pairs of either the reference type (denoted R) or alternate types $\{A,B,C\}$. Following this representation, the probability H of calling a hotspot in the position is the same as the probability to draw two instances of the same alternate type across samples. By the pigeonhole principle, this is equivalent to the probability of not having any of these four outcomes: i) all letters are R; ii) there is exactly one alternate; iii) there are exactly two distinct alternates; iv)

there are exactly three distinct alternates (Appendix Note 5 Fig 1B). Bearing this in mind, the probability to draw at least one hotspot in the position is calculated as follows:

$$H = 1 - (1-p)^N - N(1-p)^{N-1}p - 2\binom{N}{2}\left(\sum_{i\neq j} p_i p_j\right)(1-p)^{N-2} - 3!\binom{N}{3} p_A p_B p_C (1-p)^{N-3}$$

Note that this way of computing the expected number of hotspots per position yields a slight underestimation of the expected number of hotspots as per the HotspotFinder definition of hotspot, whereby at most three hotspots, corresponding to the three possible alternate alleles in the position, are possible within a single position. To account for this, only a single observed hotspot is considered per position in all calculations of observed hotspot propensities and their comparisons to the theoretical models.

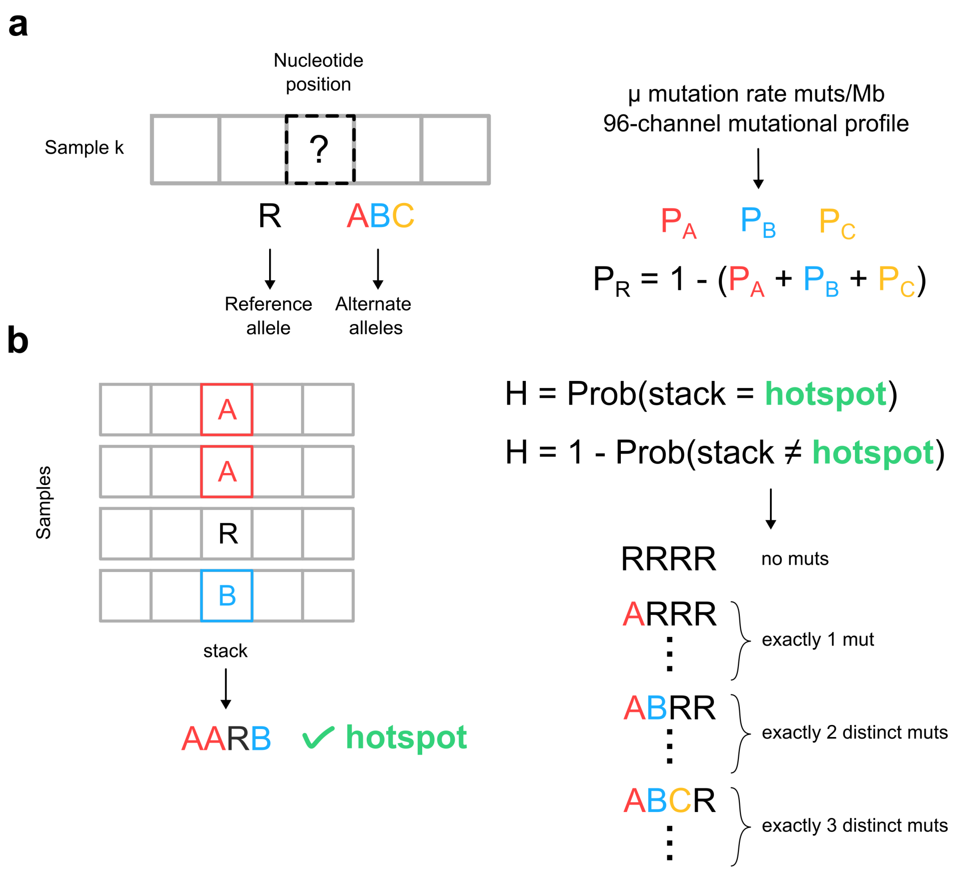

Appendix Note 5 Figure 1. Rationale behind the calculation of the expected number of hotspots in a single position. **a**) First, using the mutation rate and the profile of the mutational process operative in the samples, we infer a probability for either the reference or alternate alleles to occur in a position. **b**) With this information we can then compute the probability that the position harbours a hotspot by looking at the cases when there is not a hotspot.

3. Expected hotspot rate genome-wide

Making the simplifying assumption of a homogeneous mutation rate along the genome, we can compute the expected number of hotspots genome-wide --hotspot propensity-- generated by a mutational process $\underline{m} = (m_{t>a})$ with mutation rate μ. By the result of the previous section, note that we will only need the triplet content of the genome, i.e. the number of positions with a given reference triplet for the 32 possible (pyrimidine-centred) reference triplets. Following the computations of the previous section, if $H(t; \underline{m}; \mu)$ denotes the expected number of hotspots at a position with reference triplet t induced by the mutational process $\underline{m}$ operative with mutation rate μ, then the expected number of hotspots is given by the following expression:

$$H = \sum_t n_t H(t; \underline{m}; \mu)$$

4. Expected hotspot rate across mappable genomic chunks

Despite giving us a fair theoretical estimate, the previous calculation makes a strong assumption that is clearly violated by real data, namely, that the mutation rate genome-wide is homogeneous. However, we want to address a question that arises in our analyses: whether the expected hotspot rate diverges from the observed when we factor in the mutation rate variability across the genome.

To address this we computed the theoretical hotspot rate per megabase applying the same ideas depicted above with the precaution of keeping track of the relative mutation rate estimates of a mutational process per genomic chunk, i.e. how more/less mutable are regional chunks when compared with each other.

We proceed by inferring an empirical estimate of the relative mutability per genomic chunk and signature, i.e. using the mutations sequenced in real tumors. By means of signature deconstruction, for each sample in our cohort we can infer how confidently the mutations found in each of the 96 trinucleotide contexts are attributable to a specific mutational process (Appendix Note 3). With this measure of confidence we can then count how many mutations in a cohort match a specific mutational process (maximum likelihood (Morganella *et al*, 2016); Materials and Methods; Appendix Note 3), then map those mutation counts to each genomic chunk out of a set with high mappability (Materials and Methods). For estimation of the relative mutation rate of zero-count chunks we introduced a pseudocount consistent across chunk sizes

whereby the pseudocount added to the chunks of size s is equal to $s/1\,Mbp$: in doing so we are not changing the total mutation burden across different chunk-size analyses. With this information we can then infer the relative mutation rate attributable to a specific mutational process across chunks and apply the above described methodology to compute hotspot rates per chunk. These estimates can be finally merged to obtain the genome-wide number of expected hotspots or hotspot propensity.

5. SBS1-induced hotspot rate with a DNA methylation covariate

To study the expected hotspot propensity of signature SBS1, we built models restricted to the NpCpG>T tri-nucleotide contexts (ACG>T, CCG>T, GCG>T, TCG>T). For the three cancer types analysed (COADREAD, ESOPHA_STOMACH, NSCLC) we also retrieved DNA methylation sequencing data (Methods) from which we could derive the fold-change mutation rate between methylated and unmethylated cytosines for each NpCpG>T context. Using the relative mutagenicities of the NpCpG>T contexts derived from the SBS1 96-channel profile as a baseline (4 channels), we defined three additional SBS1 models, one for each cancer type, where two relative mutagenicity values are given for each NpCpG>T context, accounting for methylated and unmethylated cytosines (8 channels).

Just as in the previous analyses, we calculated the hotspot propensity per chunk accounting for the NpCpG sites only. Since not all the bases in the mappable genome chunks were completely annotated for their DNA methylation status, for those chunks with missing values we imputed the same proportion of methylated CpGs as the proportion found genome-wide for the same cancer type. Upon estimation of the relative mutation rate per chunk, for each cancer type and chunk size we computed the expected hotspot rate with 300 mutations/sample across 100 samples.

6. Numerical validation of the theoretical hotspot rate

To rule out implementation flaws and provide a stochastic hotspot generator consistent with the simplifying assumptions of the theoretical model, we conducted a round of numerical experiments with toy examples. Briefly, we generated cohorts of random synthetic samples for which the triplet content, mutation rate per sample, number of samples and mutational process (signature) generating the mutations were specified and we directly computed the number of hotspots for each cohort. To keep the computational cost low we resorted to small synthetic

samples ~ 1 Kbp in size. For five different configurations characterised by the triplet content of the samples (Appendix Note 5 Figure 2A) we generated 20 random cohorts with 50 samples and 10 mutations per sample, and compared the number of hotspots in each cohort against the theoretical hotspot rate. The proportion of triplets used to define each triplet content set-up were drawn randomly from the collection of mappable 1 Mbp chunks used elsewhere in this paper.

We conducted this experiment three times with signatures SBS1, SBS17b and SBS5, respectively. In all cases, the empirical hotspot rate matched closely the expected hotspot rate provided by the theoretical model of hotspot propensity (Appendix Note 5 Figure 2B) as intended.

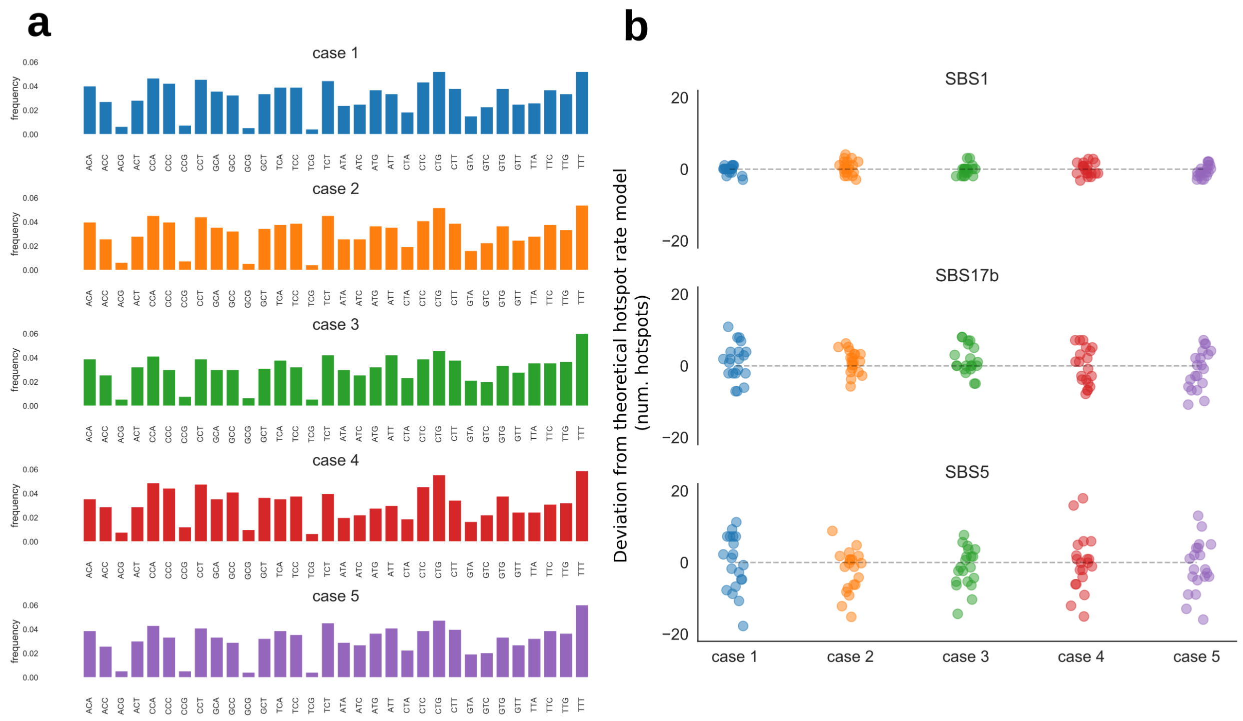

Appendix Note 5 Figure 2. Calibration of theoretical hotspot rates. **a**) Trinucleotide content frequencies of the five case scenarios considered for the random numerical experiments and **b**) centred values after stochastic simulation with respect to the expected hotspot propensity cast by the theoretical model for the three signatures SBS1, SBS17b and SBS5. In each case we produced 20 random cohorts (points in the scatter plot) using 50 samples ~ 1 Kbp in size and 10 mutations per sample.

Appendix References

Alexandrov LB, Kim J, Haradhvala NJ, Huang MN, Tian Ng AW, Wu Y, Boot A, Covington KR, Gordenin DA, Bergstrom EN, et al (2020) The repertoire of mutational signatures in human cancer. Nature 578: 94–101

Alexandrov LB, Nik-Zainal S, Wedge DC, Aparicio SAJR, Behjati S, Biankin AV, Bignell GR, Bolli N, Borg A, Børresen-Dale A-L, et al (2013a) Signatures of mutational processes in human cancer. Nature 500: 415–421

Alexandrov LB, Nik-Zainal S, Wedge DC, Campbell PJ & Stratton MR (2013b) Deciphering signatures of mutational processes operative in human cancer. *Cell Rep* 3: 246–259

An J-Y, Lin K, Zhu L, Werling DM, Dong S, Brand H, Wang HZ, Zhao X, Schwartz GB, Collins RL, *et al* (2018) Genome-wide de novo risk score implicates promoter variation in autism spectrum disorder. *Science* 362

Bergstrom EN, Huang MN, Mahto U, Barnes M, Stratton MR, Rozen SG & Alexandrov LB (2019) SigProfilerMatrixGenerator: a tool for visualizing and exploring patterns of small mutational events. *BMC Genomics* 20: 685

Cagan A, Baez-Ortega A, Brzozowska N, Abascal F, Coorens THH, Sanders MA, Lawson ARJ, Harvey LMR, Bhosle S, Jones D, *et al* (2022) Somatic mutation rates scale with lifespan across mammals. *Nature* 604: 517–524

Christensen S, Van der Roest B, Besselink N, Janssen R, Boymans S, Martens JWM, Yaspo M-L, Priestley P, Kuijk E, Cuppen E, *et al* (2019) 5-Fluorouracil treatment induces characteristic T>G mutations in human cancer. *Nat Commun* 10: 4571

C Yuen RK, Merico D, Bookman M, L Howe J, Thiruvahindrapuram B, Patel RV, Whitney J, Deflaux N, Bingham J, Wang Z, *et al* (2017) Whole genome sequencing resource identifies 18 new candidate genes for autism spectrum disorder. *Nat Neurosci* 20: 602–611

ENCODE Project Consortium (2012) An integrated encyclopedia of DNA elements in the human genome. *Nature* 489: 57–74

Frankish A, Diekhans M, Ferreira A-M, Johnson R, Jungreis I, Loveland J, Mudge JM, Sisu C, Wright J, Armstrong J, *et al* (2019) GENCODE reference annotation for the human and mouse genomes. *Nucleic Acids Res* 47: D766–D773

Goldmann JM, Wong WSW, Pinelli M, Farrah T, Bodian D, Stittrich AB, Glusman G, Vissers LELM, Hoischen A, Roach JC, *et al* (2016) Parent-of-origin-specific signatures of de novo mutations. *Nat Genet* 48: 935–939

Halldorsson BV, Palsson G, Stefansson OA, Jonsson H, Hardarson MT, Eggertsson HP, Gunnarsson B, Oddsson A, Halldorsson GH, Zink F, *et al* (2019) Characterizing mutagenic effects of recombination through a sequence-level genetic map. *Science* 363

Islam SMA, Díaz-Gay M, Wu Y, Barnes M, Vangara R, Bergstrom EN, He Y, Vella M, Wang J, Teague JW, *et al* (2022) Uncovering novel mutational signatures by de novo extraction with SigProfilerExtractor. *Cell Genomics* 2: None

Karczewski KJ, Francioli LC, Tiao G, Cummings BB, Alföldi J, Wang Q, Collins RL, Laricchia KM, Ganna A, Birnbaum DP, *et al* (2020) The mutational constraint spectrum quantified from variation in 141,456 humans. *Nature* 581: 434–443

Marco-Sola S, Sammeth M, Guigó R & Ribeca P (2012) The GEM mapper: fast, accurate and versatile alignment by filtration. *Nat Methods* 9: 1185–1188

Morganella S, Alexandrov LB, Glodzik D, Zou X, Davies H, Staaf J, Sieuwerts AM, Brinkman AB, Martin S, Ramakrishna M, *et al* (2016) The topography of mutational processes in breast cancer genomes. *Nat Commun* 7: 11383

Paulson TG, Galipeau PC, Oman KM, Sanchez CA, Kuhner MK, Smith LP, Hadi K, Shah M, Arora K, Shelton J, *et al* (2022) Somatic whole genome dynamics of precancer in Barrett's esophagus reveals features associated with disease progression. *Nat Commun* 13: 2300

Pich O, Muiños F, Lolkema MP, Steeghs N, Gonzalez-Perez A & Lopez-Bigas N (2019) The mutational footprints of cancer therapies. *Nat Genet* 51: 1732–1740

Rahbari R, Wuster A, Lindsay SJ, Hardwick RJ, Alexandrov LB, Turki SA, Dominiczak A, Morris A, Porteous D, Smith B, *et al* (2016) Timing, rates and spectra of human germline mutation. *Nat Genet* 48: 126–133

Sasani TA, Pedersen BS, Gao Z, Baird L, Przeworski M, Jorde LB & Quinlan AR (2019) Large, three-generation human families reveal post-zygotic mosaicism and variability in germline mutation accumulation. *ELife* 8

Secrier M, Li X, de Silva N, Eldridge MD, Contino G, Bornschein J, MacRae S, Grehan N, O'Donovan M, Miremadi A, *et al* (2016) Mutational signatures in esophageal adenocarcinoma define etiologically distinct subgroups with therapeutic relevance. *Nat Genet* 48: 1131–1141
