# Supplementary material for: Hotspot propensity across mutational processes
Source: Mol Syst Biol. 2023 Dec 20;20(1):6–27. doi: 10.1038/s44320-023-00001-w (PMC10883281; doi:10.1038/s44320-023-00001-w)
Supplement: Supplementary file 10 — Expanded View Figures [file 44320_2023_1_MOESM10_ESM.pdf]

Expanded View Figures

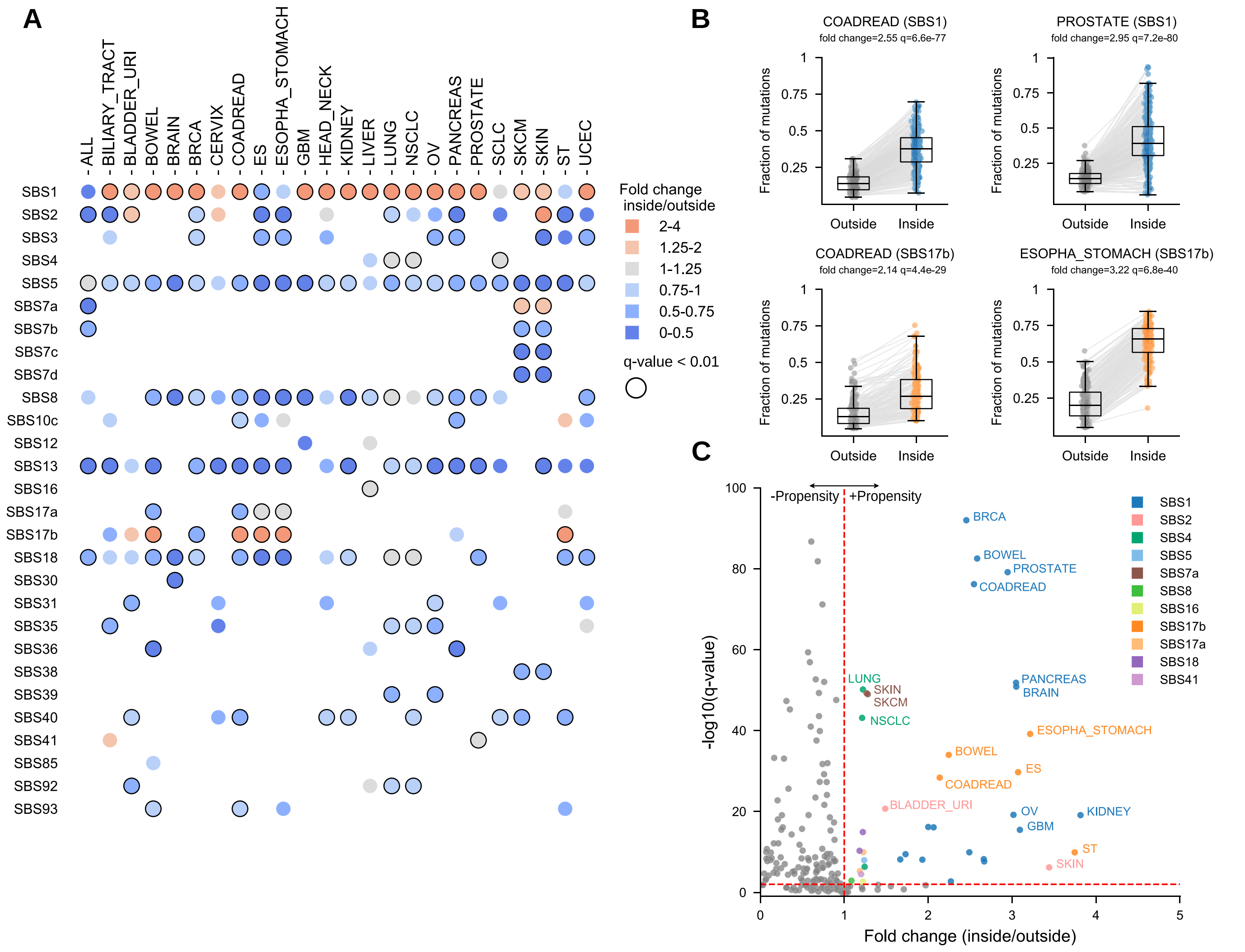

Figure EV1. Propensity of signatures to form hotspots across cancer types measured through inside-to-outside fold-change of their activity.

(**A**) Heatmap showing the fold-change of mutational frequencies inside hotspots versus outside hotspots for each active signature in a cancer type. Signatures enriched in hotspots show fold-changes greater than 1 (grey) or 1.25 (red); signatures that are depleted in hotspots show fold-changes smaller than 1 (blue). The differences in signature frequency between inside and outside mutations were compared using two-sided Wilcoxon rank-sum test followed by Benjamini–Hochberg multiple testing correction (Methods). Significant fold-changes after multiple testing correction (q-value < 0.01) are shown in bold. (**B**) Illustration of Wilcoxon rank-sum test of inside-to-outside signatures activity. Boxplots depicting signature frequencies per sample across mutations outside and inside hotspots. Boxplot centre depicts the median, and the lower and upper bounds of the box represent the 1st and the 3rd quartiles, respectively. Whiskers extend 1.5 times the IQR below and above 1st and 3rd quartiles of the distribution. Dots depict samples where the signature is active ($n = 465$ for SBS1 and colorectal cancers; $n = 486$ for SBS1 and prostate cancers; $n = 172$ for SBS17b and colorectal cancers; $n = 237$ for SBS17b and oesophageal-stomach cancers; Methods). Grey lines connect data originating from the same sample. Fold changes and q-values computed from these comparisons are shown in panels (**A**) and (**C**). (**C**) Scatter plot showing the propensity of signatures to form hotspots computed as the fold-change and significance between their activities outside and inside hotspots per cancer type. Signature-cancer type pairs showing significant differences (q-value < 0.01) and fold change greater than 1 are shown in colour.

© The Author(s)

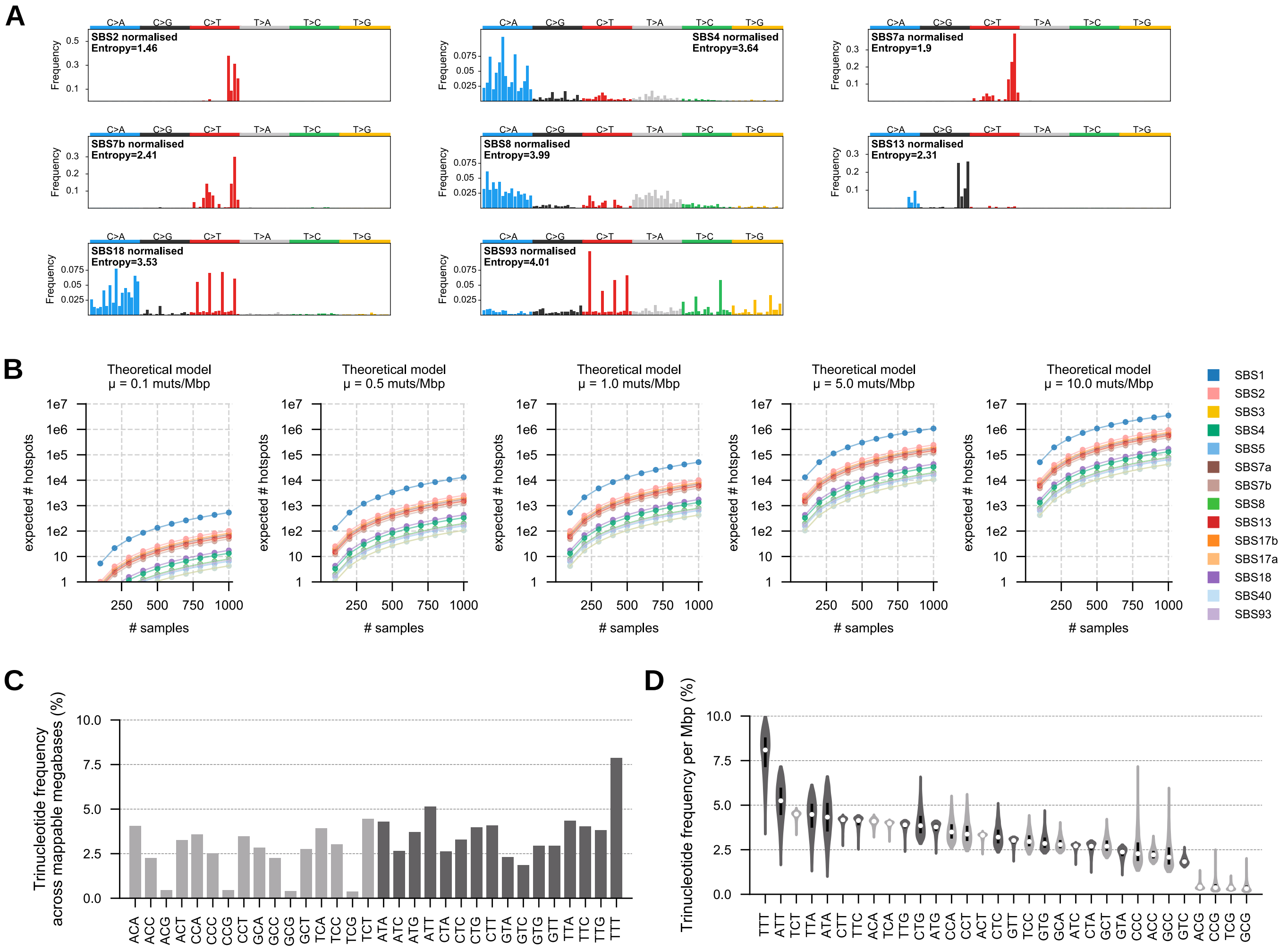

Figure EV2. Influence of trinucleotide frequencies on hotspot propensity.

(**A**) Normalised trinucleotide profiles and entropies from the 8 additional selected signatures under analysis (see Methods). (**B**) Theoretical number of expected hotspots across different mutation rates (0.1–10 mutations per sample per megabase) and sample sizes (100–1000 samples) for each of the 14 signatures. Theoretical estimates were calculated using the model of homogeneous distribution of trinucleotide-specific mutation rates across the genome (Methods; Appendix Note 5). Only positions within the mappable megabases were considered. The chosen mutation rates reflect a wide range of observed mutation rates across malignancies (Lawrence et al, 2013; Alexandrov et al, 2013a). (**C**) Bar plot showing the frequency of 32 pyrimidine-based trinucleotides across mappable genome megabases ($n = 2196$). (**D**) Violin plots showing the distribution of trinucleotides frequency across mappable megabases ($n = 2196$). White dots show the median trinucleotide frequency among megabases. Vertical black lines depict the 1st and 3rd quartiles of the distribution.

 © The Author(s)
